# Strengthening Clinical Nurses' Research Capacity: A Scoping Review of Factors That Influence Nurses' Engagement in Clinical Research

**DOI:** 10.1111/jocn.70401

**Published:** 2026-06-19

**Authors:** Lyndsay Jerusha Mackay, Laurie Lee, K. Alix Hayden, Suzanna Crawford, Gabrielle Harrison, Una Chang, Jessica Son, Nicole Chen, Joshua Yudkin, Sara Moore

**Affiliations:** ^1^ College of Nursing Texas A & M University College Station Texas USA; ^2^ School of Nursing University of Calgary Calgary Canada; ^3^ Library and Cultural Resources University of Calgary Calgary Canada; ^4^ School of Nursing Trinity Western University Langley Canada; ^5^ School of Public Health Texas A&M College Station Texas USA; ^6^ Children's Health Dallas Texas USA

## Abstract

**Aim:**

To identify and map evidence on factors that influence nurses' engagement in clinical research.

**Design:**

A scoping review was conducted in accordance with the JBI methodology.

**Methods:**

First, titles and abstracts of articles, then full‐text articles, were screened by two independent researchers against inclusion and exclusion criteria. Data was extracted into a data extraction table. Findings from articles were synthesized using thematic analysis.

**Data Sources:**

Searches were conducted in Medline All (Ovid), Embase (Ovid), APA PsycInfo (Ovid), CINAHL Plus with Full Text (Ebsco) and Scopus on April 28, 2025.

**Results:**

Seventy‐nine articles were included in this review. There were significant barriers that prevented nurses from engaging in research, including: lack of time, inadequate training, negative internal beliefs, insufficient organizational support and an unsupportive research culture. Factors that facilitated nurses' engagement in research included: research infrastructure, training and mentorship, visionary leadership, unit‐level support, improved motivational components and enhanced organizational research culture. A multi‐level framework and strategic recommendations are provided, along with an overview of the 23 interventions reported in the included articles are provided.

**Conclusions:**

Nurses are constrained from conducting research to generate nursing knowledge due to multiple barriers. Visionary leadership is needed that prioritizes nursing research, establishes infrastructure and creates a culture of research excellence to enhance the quality and safety of nursing care.

**Implications for the Profession and/or Clinical Practice:**

Healthcare organizations should support nursing research by providing nurses with paid release time from their clinical duties and building an accessible infrastructure. Infrastructure can include programmes, centres and academic‐practice partnerships that provide nurses with access to research training, experienced mentorship, librarian and statistical support, grant and manuscript writing assistance and funding pathways for small and large studies.

**Reporting Method:**

This scoping review is reported in accordance with the PRISMA‐ScR guidelines.

**Patient or Public Contribution:**

No patient or public contribution.

**Protocol Registration:**

Registered with the Open Science Framework: 10.17605/OSF.IO/5VWE9 (12/03/25).

## Abstract (highlights)

**Impact:**

- Problem addressed: A persistent gap exists between the recognized value and ethical responsibility of conducting nursing research in the clinical setting and the continued barriers nurses face that prevent them from engaging in research activities.
- Main findings: Insufficient protected time and lack of training, education and mentorship were the most reported barriers to nurse engagement in research, while key facilitators included visionary leadership, development of dedicated research infrastructure and intentional cultivation of a supportive research culture.
- Impact: Findings, including a multi‐level framework, can directly inform healthcare leaders in developing strategic initiatives and pathways to build nursing research capacity within healthcare organizations.

**What Does the Paper Contribute to the Wider Global Clinical Community?:**

- Across the globe, nurses continue to face significant barriers in the clinical setting that prevent them from engaging in research to produce evidence that directly informs their nursing care and clinical decision‐making.
- The most prominent barriers clinical nurses face that prevent them from engaging in research are a lack of paid release time and organized training and mentorship on how to conduct research.
- The findings offer a variety of practical strategies and interventions that can be implemented within healthcare organizations to increase nurses' engagement in research.

## 1 Introduction

The ability of healthcare organizations to create opportunities and pathways for clinical nurses to conduct research and generate evidence is the foundation of evidence‐based practice (EBP) and a clear marker of nursing excellence (Cato et al. 2019; Cimiotti et al. 2005). A strong research culture within a healthcare organization is essential for nurses to deliver high‐quality and safe nursing care (Harding et al. 2016; Ives Erickson and Pappas 2020). This is because nurses' increased engagement with clinical research leads to high patient and staff satisfaction, improved patient outcomes, decreased patient mortality and improved organizational efficiency (Harding et al. 2016; Shepherd et al. 2022). As well, having a hospital organization with a robust nursing research culture helps to attract and retain qualified nurses (Ives Erickson and Pappas 2020).

Nurses make up the majority of the healthcare workforce and spend the most one‐on‐one time with patients and families, providing direct care and implementing research interventions on the front lines (Powell 2015). Thus, they have a unique perspective to recognise when system policies or nursing care workflows fail patients, families and populations (Powell 2015). This positions them to ask the most important and relevant research questions that can directly impact care delivery and patient outcomes. Therefore, nurses should be encouraged and equipped to ask questions about their practice and supported in conducting research that identifies innovative solutions to clinical problems (Ives Erickson and Pappas 2020).

Nursing research includes the systematic inquiry that generates new knowledge and evidence, which can then be evaluated and disseminated to inform nursing practice and advance the profession (Mulkey 2021). Research can begin with a literature review to highlight gaps, then proceed with data collection and analysis, followed by the reporting of study results and interpretations to the healthcare community (Ives Erickson and Pappas 2020; Polit and Beck 2020). EBP includes searching, retrieving, appraising, synthesizing and incorporating current high‐quality evidence with patient values and preferences, including context, into clinical practice to improve nursing care (Cline et al. 2017). EBP is considered a problem‐solving approach to the delivery of nursing care that incorporates the best evidence from high‐quality research studies into the delivery of everyday nursing care (Camargo et al. 2018). It is well‐established within the profession of nursing that to improve patient outcomes, nursing care must be based on the most current evidence (Twelvetree et al. 2019). For nurses to have this evidence upon which to base their practice, they must be granted the opportunity to readily engage in clinically oriented nursing research to produce and develop clinical evidence that they can use to guide their care decisions (Rivera and Shelley 2024; Twelvetree et al. 2019).

Nurses have a professional responsibility to incorporate quality evidence into their practice when it exists and also to generate evidence to answer clinical questions and fill gaps where evidence does not exist (Alomari et al. 2020; Ives Erickson and Pappas 2020). According to the American Nurses Association (ANA), nurses have an ethical obligation to engage in knowledge development, research and scholarly inquiry. Under Provision 7.1 of the ANA Code of Ethics for Nurses, nurses should engage in knowledge production to inform nursing practice, using a diversity of methodologies and ways of knowing to contribute to nursing knowledge (American Nurses Association 2025).

Even though nurses have a professional and ethical responsibility to conduct research, thereby enabling patients to receive high‐quality, safe care that results in improved outcomes, clinical nurses face significant barriers and are rarely given the opportunity to fully engage in clinical research (Henshall et al. 2023). Nursing is considered to be an academic discipline, but the infrastructure that supports nurses' engagement in research is variable and most often underdeveloped (Shepherd et al. 2022). Clinical nurses rarely perform clinical research (Amicucci et al. 2022) because they face significant barriers that prevent them from conducting nursing‐focused research studies (Arthur et al. 2019; Bench et al. 2019; Duffy et al. 2016). Therefore, there is a significant gap: nurses and organizations recognise the value and professional responsibility of nurses' engagement in clinical research, yet nurses are rarely supported to do so. To address this gap, it is important to identify barriers that prevent nurses from engaging in research, as well as facilitators that could be implemented within programmes and initiatives to increase nurses' successful engagement in research.

## 2 Aim

To identify and map evidence on factors that influence nurses' engagement in clinical research.

## 3 Methods

### 3.1 Study Design

A comprehensive scoping review was conducted to map and identify factors that influence clinical nurses' engagement in research and/or EBP. This scoping review followed the JBI methodology for scoping reviews (Peters et al. 2024), adhered to the Preferred Reporting Items for Systematic Reviews (PRISMA‐ScR) checklist (see File S1) (Tricco et al. 2018) and has been registered with the Open Science Framework (registration number: osf.io/5vwe9). Given the substantial number of included articles (*N* = 286) and the important distinction between nursing research and EBP activities, the research team strategically organized the included articles into three categories, articles addressing factors that influence nurses' engagement in: (a) research, (b) EBP and (c) both research and EBP activities simultaneously. This categorisation reflects the fundamental difference between nursing research (the systematic generation of nursing evidence through inquiry) and EBP (the utilisation and implementation of existing evidence into clinical practice). By analysing these articles separately according to their grouping, the team aimed to identify both differences and similarities in the barriers and facilitators affecting nurses' engagement in research versus EBP activities, while also highlighting how they are synergistically integrated. This paper reports findings from articles on factors that influence nurses' engagement in research.

### 3.2 Search Methods

Following Peters et al. (2020) guidelines, an initial search was conducted in Google Scholar to identify seed articles that met all the inclusion criteria and triggered none of the exclusion criteria. Next, the relevant keywords related to ‘engagement’ were identified in the titles and abstracts of the seed articles. These keywords, along with keywords associated with evidence‐based practice or research, were used to develop a search strategy in Medline All (Ovid). In addition, the search strategies from the reviews conducted by Boaz et al. 2015 and Chalmers et al. (2023) were adapted and incorporated into the final search strategy for this review. Prior to database translation, the final Medline search was pilot tested with the seed articles to ensure that all were captured by the search.

The final search utilized proximity searching in order to focus results on nurse engagement with EBP or research. Keywords for barriers or facilitators were not incorporated to ensure that the search was comprehensive and articles would not be missed.

Databases searched included: Medline All (Ovid), Embase (Ovid), APA PsycInfo (Ovid), CINAHL Plus with Full Text (Ebsco) and Scopus. The search was the same for all databases, except for the proximity operator, which was translated to be appropriate for the database platform. The search was not limited by date or language. The final searches for all databases are available in File S2.

### 3.3 Inclusion and/or Exclusion Criteria

#### 3.3.1 Population

The population included in this review article was nurses, including registered nurses, nurse practitioners, licensed practical nurses and advanced practice nurses, who work within the clinical setting. Nursing attendants and nursing aids were excluded. Nursing students enrolled in a baccalaureate programme were excluded; however, nurses enrolled in a graduate degree programme were included. Articles that included physicians and allied healthcare professionals were included if > 50% of the sample size were nurses.

#### 3.3.2 Concept

The concept of this review was factors, including barriers and facilitators, that influenced nurses' ability to engage in research activities. Nursing research was defined as the systematic inquiry that generates new knowledge and evidence, which can then be evaluated and disseminated to inform nursing practice and advance the nursing profession (Mulkey 2021; Polit and Beck 2020). Therefore, articles had to discuss factors that directly enabled or inhibited nurses' engagement in the research process (i.e., attaining research training and mentorship, developing a research proposal, collecting data, analysing data, writing findings and manuscripts and disseminating study findings). Articles that included nurses' engagement in the EBP process were not included in this review paper but will be reported in a future manuscript.

#### 3.3.3 Context

The context was limited to inpatient care units, including: intensive care units, emergency departments, oncology units, mental health units, neonatal intensive care units and outpatient care units. Outpatient care clinics were included as long as they were affiliated with a tertiary care hospital. Community health, public health, physician offices, rural health and academia were excluded. Articles reporting research studies were limited to those conducted in developed countries defined by the United Nations Department of Economic and Social Affairs (United Nations 2025). See File S3 for list of 37 countries. This restriction was used to ensure the included articles reported findings from healthcare systems with resource levels, workforce structures and regulatory expectations similar to those in North America. Review articles conducted by authors living in any country were included.

#### 3.3.4 Types of Sources

Qualitative, quantitative and mixed‐methods studies were included, along with reviews (e.g., systematic review, meta‐analysis, literature review and scoping review), project descriptions, quality improvement studies, discussion papers and case studies published in English in 2010 or after. A date limiter was applied to the publication date during full‐text screening as decided by the research team for the following reasons: (a) to keep the final number of included studies manageable, (b) to ensure the articles reflected contemporary models of healthcare delivery and (c) to ensure the articles captured the current conceptualisation of nursing clinical research. Grey literature, including government reports, dissertations, theses, editorials, opinion pieces and conference abstracts, was not searched or included.

### 3.4 Study Selection

All titles and abstracts from the search were uploaded into Covidence (Veritas Health Innovation 2020) and duplicates were removed. The inclusion and exclusion criteria were pilot tested on 50 randomly selected titles and abstracts until the reviewers reached a minimum inter‐rater reliability of 90%. All titles and abstracts were screened by two independent reviewers (UC, LM, JS) based on the inclusion and exclusion criteria. Articles that were deemed included were retrieved in full text and screened again in their entirety by two independent reviewers and reasons for exclusion were recorded. Any discrepancy at any phase of screening was resolved by discussion. See Figure 1 for the results of the search and screening process reported in a PRISMA‐ScR (Tricco et al. 2018).

**FIGURE 1:**
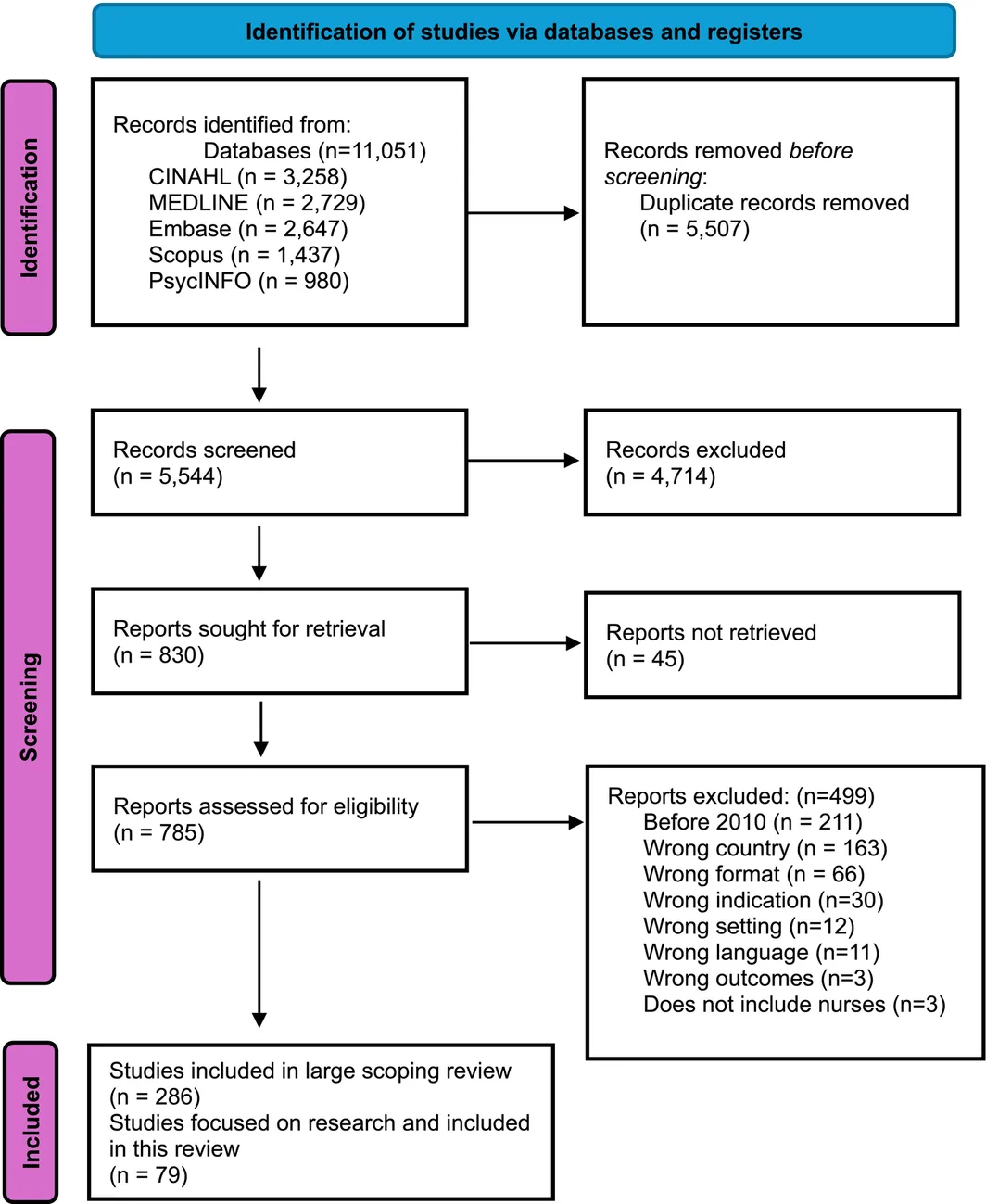
PRISMA flow diagram of source identification for scoping review. [Colour figure can be viewed at wileyonlinelibrary.com]

### 3.5 Data Abstraction

Data from the included articles were extracted into a table adapted from the JBI data extraction instrument (Peters et al. 2020). The data extraction table was pilot tested on the first 10 articles that appeared in alphabetical order as per the authors' last name. Elements of the data extraction table included: authors, date, country, aim/purpose/research question(s), sample with participant characteristics, methods/study design, intervention (if used), measurement tools/theoretical framework (if used)/outcome measures, barriers, facilitators, overall findings, recommendations and outcomes. Quality appraisals were not conducted because they are not required for the JBI scoping review methodology (Peters et al. 2020).

### 3.6 Data Analysis and Presentation

The articles have been compiled and are presented in Table 1 with an overview of the findings from each one. The extracted data from the findings were analysed using thematic analysis (Braun and Clarke 2006). First, data were coded by placing labels on segments of data. Then, the codes were collated and grouped into categories based on similarities. Categories were then collapsed into themes and a description of each is provided.

**TABLE 1:** Summary of included articles.

Author, date, country	Aim/purpose/research question	Sample with participant characteristics	Method/study design	Intervention	Measurement tools/theoretical framework used/outcome measures	Main results: barriers	Main results: facilitators	Overall findings/recommendations/outcomes
Akerjordet et al. (2012a, 2012b), Norway	To examine clinical nurses' research capacity and related factors (i.e., the different phases of the research process).	Registered Nurses (RN) (*N* = 364) working in a Norwegian university hospital throughout clinical care.	Quantitative descriptive	N/A	Australian questionnaire that was translated to Norwegian	Lack of research skills (inter‐rater reliability, pilot test instruments, identify confounding variables) and limited financial support and funding.	Seen purpose of research, access to relevant literature, research education and training, collaboration and mentorship, prior research experience that helps them to feel confident.	Recommendations: Increased support from nurse managers to create an environment that inspires nurses, development of research and innovative nursing practice, consider organizational structures and resources, enhance nurses' research skills, increase nurse's time they are involved in research.
Akerjordet et al. (2012a, 2012b), Norway	To determine clinical nurses' interest in and motivation for research and identify management and organizational resources to improve nurses' research capacity in practice.	RNs (*N* = 364) clinical nurses from a Norwegian university hospital in both inpatient and outpatient settings	Quantitative descriptive	N/A	Research Needs Analysis Survey	Lack of designated time, interest and knowledge constituted important research barriers as well as lack of research supervision and support.	Research supervision, identification of an actual research area, funding, support for the preparation of a grant proposal, literature search, available opportunities and adapted working hours, role models.	Recommendations: Develop an organizational climate that values to support nurses' research engagement. Nurse managers are integral stakeholders in supporting research, must be aware and competent of nursing research. Awareness of organization characteristics and visionary leadership, research supervision.
Alomari et al. (2020), Australia	To explore the outcomes of active participation in an action research project on building the research capacity of clinical nurses.	RNs (*N* = 6) at an Australian paediatric teaching hospital	Qualitative action research	Active participation in an action research project on building the capacity of clinical nurses	Nurses actively participated in the research process of an action research project by being part of an Action Research Team (ART).	Initial feelings of being overwhelmed by data, fear of lack of research knowledge, negative perceptions of research based on prior academic experiences, apprehension about peer response and role clarity.	Mentorship and support from experienced researchers, structured research training and resources, respectful and collaborative team environment, direct application of research to clinical practice, sense of ownership and value in their contributions, incremental successes that built confidence and sustained engagement.	Outcomes: Nurses became aware of flaws in their practice, recognized a need to improve practice and develop new personal qualities. They experienced increased self‐esteem as they led to practice changes and became aware of the lengthy research process, becoming curious and motivated to improve other areas of their practice. Recommendations: engage nurses in applicable research with experienced researchers. Take collaborative and participatory approaches. Establish infrastructure necessary to engage nurses in research.
Aloweni et al. (2017), Australia	To develop research competence and promote evidence‐based practice (EBP) among clinical nurses.	RNs (*N* = 4) with 6 years of clinical experience; selected based on research interest and clinical background	Programme description	Nursing research immersion programme	Open ended questions were asked of the nurses regarding the programme	Lack of time, lack of support and limited research knowledge.	Protected time from clinical duties (1 day/week), one‐on‐one mentorship by experienced researchers, topic alignment with clinical roles, formal selection and interview process to ensure readiness and relevance, organizational support from nurse managers and senior leadership.	Prior programmes (e.g., EBP workshops) showed poor translation to practice, built research competence among clinical nurses and developed first generation nurse scientists.
Amicucci et al. (2022), Italy	To describe the level of participation in research activities over the last 10 years in a sample of nurses and allied health professionals (AHPs) working in an Italian academic tertiary paediatric hospital; and to examine which socio‐demographic and professional characteristics are associated with these tasks.	Nurses and AHP (*N* = 921) working in all five settings of the paediatric hospital, including staff, manager, research nurse or AHP on duty	Quantitative descriptive	N/A	17‐item survey covering education, roles, research activity, mentorship, barriers, facilitators	Language prevented them from reading international journals and writing scientific papers, gender roles as a female.	Male gender, working as an AHP, manager or clinical specialist, having a master's degree, attending a specific course about research within the hospital, participation at the internal hospital research group, regular reading of scientific journals, working in research laboratories or outpatient clinics.	Recommendations: Identify strategies to promote research (e.g., protected time, specific education, strengthened collaboration with academics, financial support); managers should support development of research culture.
Arthur et al. (2019), United States	To assess nurses' attitudes and perceptions about research.	RNs (*N* = 623) employed at a National Cancer Institute	Quantitative descriptive	N/A	Nursing Research Attitude Scale, Research Environment Scale, Research Involvement Survey	Time to conduct research, lack of knowledge and availability of research‐related resources, need for protected time, financial support, education and collaboration for nurses to actively participate in research.	Advanced degrees and/or promotion to administrative and other indirect care roles, additional education/training related to research, workshops, partnering nurses who have research questions with doctoral prepared nurse scientists.	Outcomes: Nurses research is evidence of nursing excellence for maintaining Magnet status. Recommendations: Increase nurses' access to mentors and resources, offer research fellowship programmes to offer protected time off and didactic content and the development of clinical‐academic partnerships.
Back‐Pettersson et al. (2013), Sweden	To describe clinical nurses' experiences of participating in a Research and Development (R&D) programme and its influence on their interest and ability to conduct and apply nursing research.	RNs (*N* = 12) in Sweden with a bachelor's degree in nursing pursuing a master's degree	Qualitative descriptive study	Research & development programme	Data were collected in focus group interviews. After 1 year content analysis was used to analyse the data	Tensions between academia and clinical practice (e.g., differing expectations, supervision models), lack of opportunities in some workplaces to implement project findings, feelings of isolation from colleagues not in the programme.	Supportive leadership and structured mentorship from senior researchers, integration of academic education and workplace projects, group‐based learning and peer support, protected time for study and project work, opportunities for presentation and dissemination of research.	Recommendations: Strong leadership, organizational and supportive infrastructure, explicit research strategies and programmes, collaboration between academic leaders and clinical institutions.
Bench et al. (2019), United Kingdom	To explore orthopaedic nurses' views regarding research priorities in neuro‐musculoskeletal care and the perceived barriers and facilitators to engage in research.	Orthopaedic RNs (*N* = 75) at a UK specialist hospital	Mixed‐methods	N/A	Questionnaire of five closed questions on academic qualifications, experience of research and future aspirations., focus group interview	Research not perceived as part of nurses' roles, time constraints, shift patterns, burnout, lack of confidence, skills, academic background, fear of research terminology/language, lack of visibility, support, leadership and funding, disempowerment and hierarchical barriers.	Strong leadership and positive research culture, research training, mentorship, workshops and shadowing, inclusion of research in appraisals and revalidation, peer support, journal clubs, internal conferences, protected time, flexible training formats, visible research staff.	Recommendations: Develop effective leaders who can create positive research culture across the organization and work collaboratively to address research resources and educational needs.
Berthelsen and Holge‐Hazelton (2015), Denmark	To determine the self‐perceived theoretical knowledge and practice research competencies among orthopaedic nurses and their interest and motivation to increase these in everyday practice.	Orthopaedic nurses (*N* = 87)	Quantitative descriptive	N/A	Adapted Research Needs Analysis Survey	Lack of confidence in their knowledge of research, lack of time, lack of interest, self‐perceived lack of ability to perform research, lack of acceptance from others, lack of guidance or teaching.	Interest/understanding of the importance of research, time built in for provided classes or learning, mentorship, benefits for participating in research.	Recommendations: Develop an educational intervention that focuses on supporting orthopaedic nurses' requests for more evidence‐based knowledge and practical research competencies.
Berthelsen and Holge‐Hazelton (2017), Denmark	To report an analysis of the concept of nursing research culture in the context of clinical nursing practice.	N/A	Concept analysis	N/A	N/A	Lack of organizational resources, the mental separation of research and clinical practice, lack of support and acceptance from colleagues and low confidence in research ability.	Emphasis on the importance of nursing research, provided time and education towards research, funding for research staff to help facilitate and accepting culture within the staff.	Outcomes: Leaders must be prepared to emphasise the importance of research and provide time and financial resources. A nursing research environment is required to increase EBP.
Berthelsen and Holge‐Hazelton (2017), United Kingdom	To report an analysis of the concept of nursing research culture in the context of clinical nursing practice.	Papers from a literature search (*N* = 10)	Concept analysis	N/A	Action research approach within an acute NHS hospital.	Lack of time, resources and organizational support, limited academic training and research competencies among clinical nurses, managerial resistance or indifference, lack of shared language or cultural alignment around research, infrequent socialisation into a research culture for new staff.	Visible, passionate and qualified leadership, academic mentorship, journal clubs, structured training, positive collegial relationships and shared research values, protected time, financial support and institutional endorsement of research, inclusion of research in mission statements and strategic goals.	Although the method of concept analysis has been criticized and heavily debated, the development of nursing research cultures based on the defining attributes and antecedents of the concept will be important to emphasise evidence‐based clinical nursing care. Further research should support the development and the implementation of nursing research culture in clinical nursing practice.
Berthelsen and Hølge‐Hazelton (2016), United Kingdom	To describe the orthopaedic nurses' experiences regarding the relevance of an educational intervention and their personal and contextual barriers to participation in the intervention.	Orthopaedic nurses (*N* = 32) in a Danish regional hospital	Mixed methods	N/A	21 item questionnaire and two focus group sessions.	Prioritizing leisure time, maintaining their responsibilities to prevent overburdening their colleagues, uncomfortable leaving the section.	The top facilitator was the interest in learning.	Recommendation: To ensure success and increase nurse participation in research, education and educational sessions should be integrated into daily clinical practice.
Berthelsen and Holge‐Hazelton (2015), Denmark	To determine the self‐perceived knowledge, competencies, interest and motivation regarding research among orthopaedic nurses in an orthopaedic department at a Danish Regional Hospital.	Orthopaedic RNs (*N* = 43) at the Regional Hospital of Zealand, Denmark	Mixed method	N/A	Questionnaire with 24 questions	Lack of time, lack of perceived ability, lack of acceptance from colleagues and head nurse to prioritise research, lack of teaching and guidance.	Desire to increase knowledge, support from head nurses, support from colleagues, clinical guidance, having a role model.	N/A
Bohman et al. (2013), Sweden	To explore nurses' perception of nursing research and its implementation in a clinical context.	RNs (*N* = 16) working in a secondary care setting	Qualitative descriptive study	N/A	Focus groups, interviews which were recorded and transcribed.	Lack of time, limited research knowledge, insufficient support from management and value of nursing research from the organization, lack of access to research articles, perception that research is not relevant to daily work and negative attitudes towards research, implementing research can be cumbersome.	Positive attitudes towards research, higher education level, prior research involvement, managerial encouragement, access to research literature and opportunities for continuing education.	Outcomes: A more holistic view of the patient and uniqueness in each caring encounter. Research led to better treatment options and raised the standard of nursing care. Recommendations: Increase RNs' education on research, in‐service training on basic research methods, nursing training programmes that have an introspective approach.
Brown et al. (2018), United Kingdom	Aims to explore the views of nurses towards child health research and to identify factors influencing their willingness to facilitate it in practice.	RNs (*N* = 105) and midwives in three neonatal and two children's units in two discrete acute hospital sites	Quantitative descriptive	N/A	Questionnaire developed for the study	Worry about not implementing the protocol correctly, overburden on family during stressful times, difficulty keeping up to date with research protocols, legal concerns if something goes wrong, workload difficulties, clinical research protocols are confusing, no incentive to participate, not given the option to refuse participation, belief children should not be used as participant of research, negative attitudes of other staff, belief clinical research is boring, belief clinical research is not the role of nurses, additional paperwork, ethical concerns (e.g., parent consent, treatment equity).	Recognise the importance of research for child specific treatment, adequate training/education and allocated time, recognition for research involvement, research that is relevant to practice, protocols made accessible and easy to understand, research that improves patient care and communicative research outcomes.	Most participants (995) supported research involvement in the clinical area. Motivating factors of research: desire to improve patient care, benefits to the patient, contribute to EMP. Recommendations: training and education, managerial support, engaging clinical staff and communication of research activities.
Carter et al. (2020), United States	To describe the programmatic details and the outcomes of a competitive, two‐year Academic‐Practice Research Fellowship for clinical nurses.	Nurses (*N* = 13) within a research fellowship	Case study	Academic‐Practice Research Fellowship	Program evaluations through review of administrative records	Lack of time, limited research knowledge/skills/confidence, organizational restrictions (e.g., PI eligibility).	Protected research time, one‐on‐one mentorship, statistical support and manuscript‐writing workshops, monthly fellowship meetings and EBP/research committee participation.	Outcomes: 13 nurses have been accepted into the program; all nurses in the first graduating class have completed a research project, presented and 80% published.
Chlan et al. (2020), United States	To describe two scholar programs that provide nurses with mentored opportunities to ultimately build an internal pipeline of practice‐based nurse scientists.	Clinical registered nurses in a large U.S. Magnet designated health system	Case study	Clinical Nurse Scholar Programme	N/A	Limited time (only 1 day/week for research), need for sustained internal financial support, balancing clinical coverage when nurses are away for scholar activities, potential confusion between roles of PhD advisors and internal mentors, limited research training in undergraduate curriculum, difficult sustaining funding, disseminating findings, limited time results in smaller project, insufficient mentoring capacity.	Leadership support from Associate Dean for Nursing Research and CNO, structured mentorship and scheduled meetings with nurse scientists, protected time and clear programme metrics, philanthropic and internal funding, opportunities for dissemination and professional networking, linkage of programme outputs to Magnet designation exemplars.	Outcomes: Increased nurse consideration to pursue nursing research career path, increased research training, opportunities for publishing and presenting findings.
Colwill et al. (2024), United States	To examine the association between personal, professional and work characteristics of nurses and their research capacity in the clinical setting.	RNs (*N* = 310) within a midwestern and southern United States healthcare system	Quantitative descriptive	N/A	Survey developed for the study	Lack of time and competing clinical priorities, limited research skills and experience, minimal exposure to research opportunities in daily practice, perception that research is not part of the clinical nurse role.	Access to mentors and role models, organizational and team support for research, opportunities for research training and professional development, integration of research expectations into clinical roles, frequent exposure to research, experience coaching newly hired nurses, experience with nursing research, protected time, receiving communication about research opportunities, meeting with mentor, journal club participation, having a curiosity, high personal locus of control, support from work colleagues and nurse friends, non‐direct patient care positions, delivering local, regional, national presentations, had family members or were personally hospitalized.	Outcomes: Higher levels of research engagement are significantly associated with psychological traits like curiosity and grit, as well as professional support systems, rather than personal demographics like age or experience.
Crozier et al. (2012), United Kingdom	To develop sustainable resources and activity in one hospital to encourage, support and build research activity and innovation among nurses and midwives.	Study steering group members (matrons, specialist nurse, education manager, R&D officer, three project leads)	Qualitative action research	The Innovations in Nursing and Midwifery Practice Project (INMPP)	Interviews and analysis	Lack of time and workload pressures, perception of research as a medical activity, strategic thinking and resource planning skills lacking in junior staff, resistance to practice change from some staff.	Strong, visible leadership from the hospital and the university, mentorship and coaching from matrons and senior staff, dedicated funding and resources for project time, structured support (workshops, templates, proposal guidance), partnership between hospital and the university, alignment with nationally driven agendas for clinical care improvement.	The development process takes time and sustained effort. Leadership skills, resources and time management are required. Nursing research capacity can be established where there is enthusiasm to drive the agenda.
Davis et al. (2019), United States	To describe the development, structure and outcomes of a decade‐long academic practice partnership between a school of nursing and a health system to build and sustain nursing research capacity.	Clinical nurses, nurse leaders and advanced practice nurses at The Queens Health System (Honolulu, Hawaii), along with faculty and graduate students from the University of Hawaii at MÄnoa School of Nursing & Dental Hygiene	Qualitative observational study	Academic‐Practice Partnership	Administrative records of approved projects, completion status, dissemination outputs, mentoring activities	Lack of protected time for clinical staff to engage in research, limited prior research experience among nurses, ongoing need for sustained funding and leadership support.	Strong leadership commitment from both academic and practice settings, formalized research infrastructure and policies, mentorship and consultation from experienced nurse researchers, funding for projects and dissemination, alignment of research with organizational priorities and patient outcomes.	Outcomes: 28 approved studies, 17 completed, five in progress, 3 publications, 23 podium presentations, 8 poster presentations. Successfully bridged education and practice in the community.
Drury et al. (2024), International	To determine research knowledge, attitudes and practices among cancer nurses and understand factors influencing nurses' involvement in research.	RNs (*N* = 366) at Memorial Sloan Kettering Cancer Centre	Quantitative descriptive	N/A	Nursing Research Knowledge, Attitudes Survey and Barriers to Nurses' Participation in Research Questionnaire	Lack of time, lack of incentive/reward, lack of knowledge/skills (submitting an ethics application and funding application, setting up study procedures, finding data collection too, collaborating with the office of nursing research), lack of training opportunities, lack of experienced mentors.	Clear pathway to receive mentorship, lack of time, having clinical time to participate in research, incentives, holding a specialist, advance practice or administrative role, graduate qualification, authoring a paper, previous experience conducting research and submitting a grant.	Recommendations: Organizations must have strategies to overcome barriers to research in practice. One way is to invest in research fellowships that build capacity (e.g., knowledge and confidence building, preparing research protocols, IRB submissions, analysis, dissemination). Investment is needed to overcome barriers and address education, research culture and resourcing to support nursing research. Outcomes: Less than 15% were involved in research. Participants expressed a desire to participate in research.
Duffy et al. (2015), United States	To report baseline findings from a research focused academic service.	Nurses (*N* = 75) in an academic medical centre in the Mid‐Atlantic Region	Quantitative descriptive	N/A	Demographic tool, Evidence based Practice Attitude Scale (EBPAS), Evidence Based Practice Confidence (EPIC) scale, Evidence Based Practice Questionnaire	Low research knowledge among staff and leaders, limited confidence in EBP activities, lack of embedded research activities in nurse leader roles.	Positive attitudes towards EBP, Higher education and professional certification are associated with higher confidence and knowledge, Academic‐Service Partnership (ASP) framework offering embedded scientists, shared scholarly culture and EBP teams.	Recommendations: To shift from traditional education to experiential learning and actively supporting professional certification to boost nurses' confidence and competence.
Dupin et al. (2014), United Kingdom	To report from a study aimed at illuminating how French registered nurses experience and engage in nursing research in clinical practice.	RNs (*N* = 7) from the wound care team, clinical research team and pain control team	Qualitative ethnographic study	N/A	Participatory observations, field notes and interviews	Time pressures, workload, limited formal support for integrating research into practice, lack of continuity for embedding changes, organizational resistance to change.	Relevance of research to clinical concerns, supportive leadership and research mentors, opportunities for hands‐on involvement in research activities, perceived link between research and professional development/credibility, critical thinking and team‐management skills, clinical expertise, access to education.	Findings: Nurses developed a feeling of belonging to the research over time, through shared practices and experiences.
Engh et al. (2022) United States	To describe the implementation and outcomes of a hospital‐based endower nursing research chair model as a strategy to foster interdisciplinary engagement and collaboration in clinical research.	Clinical nurses, advanced practice clinicians, physicians, operational leaders, experienced scientists and first‐generation researchers	Qualitative case report	Hospital‐based endowed chair in nursing research	N/A	Time constraints, lack of resources, intimidation of the research process, lack of knowledge, lack of understanding how to write a research question, prepare a protocol, submit for ethics approval, secure funding, grant writing and activate the study.	Endowed nursing research chair funding and leadership, protected time for Conway Nursing Research Scholars, accessible, central location and online participation options, alignment with hospital priorities, unit‐based champions and continuing education credits.	Recommendations: Identify and recruit the scientific and clinical resources, take active leadership roles that visibly support and communicate the benefits of participating, role model participation, recognise scholarship contributions and create staffing resources.
Evans et al. (2014), Australia	To explore perceptions of registered nurses participating in research activities at a metropolitan hospital and identify barriers and facilitators to engagement.	RNs (*N* = 10) from a large metropolitan hospital in Western Australia	Qualitative interview research	N/A	Field notes from 9 months of participant observation, audio‐recorded semi‐structured interviews, documentary analysis of research outputs and organizational records	Time constraints (busy personal lives) and workload, limited research knowledge and skills, lack of formal research support structures, competing clinical priorities, negative research culture, not understanding research was within the nursing role.	Mentorship and guidance from experienced researchers, access to research training and resources, organizational support and recognition, clinically relevant research topics, communication, opportunities to undertake research activities, witnessing personal benefits from research engagement, graduate nursing education, gaining experience, improving research culture, protected time, improving appraisal skills.	Nurses believed that nursing research would result in high quality nursing care.
Farquharson (2024), United Kingdom	To explore the perceptions of nurse lead‐investigators about what has helped or hindered them in leading funded research projects.	Nurse lead investigators (*N* = 36) from major UK research funders	Quantitative descriptive	N/A	Online survey answering 2 open ended questions	Lack of structured clinical academic career pathways, insufficient protected time, heavy teaching/clinical workloads, negative or obstructive attitudes from nursing colleagues, perceived lower value of nursing research, underestimation by other disciplines.	Access to mentors with a track record in securing funding, multi‐disciplinary collaborations, research‐intensive environments, strategic thinking beyond patient‐level interventions and protected time.	Outcomes: Leading funded research depends heavily on multi‐disciplinary collaboration and experienced mentorship, while significant barriers include a lack of protected time and horizontal aggression from nursing colleagues.
Fowler and Brown (2020) Australia	To explore nurses' experiences of actively participating in an action research project within a residential early parenting service.	RNs (*N* = 21), all female, from three residential early parenting units in New South Wales	Qualitative action research	N/A	Five focus groups with semi‐structured interview questions, thematic analysis (Braun & Clarke) to identify major themes and subthemes	Initial lack of interest/ownership, poor communication about project purpose and expectations, lack of communication about research projects to staff, perception of research as an imposed task, high workloads and time constraints, unequal contribution across staff, poor research culture.	Visible leadership from unit practice development (UPD) facilitators, clear communication and information tools (e.g., info sheets), integration of research tasks into daily workflow, observational activities fostering reflection and peer learning, tangible positive feedback from parents validating nursing care, visible outcomes of research that are applicable to nurses, learning about the research process, a sense of ownership, research findings improving practice.	Outcomes: Participants reported increased confidence and engagement over time.
Fullam et al. (2018), United Kingdom	To describe and evaluate the Research Excellence Across Clinical Healthcare (REACH) programme among clinical staff in a large Irish hospital group.	RNs, midwives and allied health professionals from diverse clinical specialties, employed in hospital group settings (*N* = 17)	Mixed methods	Research Excellence Across Clinical Healthcare (REACH)	Programme metrics: number of nurse fellows, IRB‐approved studies, grants, abstracts, manuscripts, conference presentations and training reach	Clinical workload and time constraints limit participation, variable managerial support across departments and limited pre‐existing research skills in some participants.	Protected research time funded by the programme, structured mentorship from experienced researchers, multi‐disciplinary peer learning environment, organizational commitment from hospital group leadership.	Outcomes: Individual (*N* = 17) and small group (*N* = 4) research groups were supported. Increased research dissemination in journals, posters, oral presentations at national/international conferences. Three went on to do PhD and 5 projects are ongoing.
Gallagher et al. (2021), United Kingdom	To determine neonatal nursing‐led research activity within the UK and identify barriers and facilitators to such research.	Neonatal RNs working at or towards a Masters or Doctoral qualification in the UK (*N* = 56)	Quantitative descriptive	N/A	Web based survey	Lack of time for research, limited funding knowledge/access, few role models/mentors from neonatal nursing, limited colleagues and institutional support.	Organizational and managerial support, positive research culture in clinical settings, dedicated facilitators and mentors, networking and collaboration opportunities.	Recommendations: Development network of research‐active nurses, harness social media to engage nurses, develop an informal network of mentors and centralise arch information online.
Gettrust et al. (2016), United States	To describe processes for exploring and implementing an academic clinical research partnership, engaging nursing staff in research and sustaining enthusiasm throughout the study.	Frontline nurses from the orthopaedic clinic, operating room and postsurgical units (*N* = 41) at Clement J. Zablocki VA Medical Centre	Case study	N/A	Observed staff engagement, collaboration, integration of research activities and positive attitudes	Initial lack of awareness or familiarity with the research process among clinic staff, potential for disruption to clinic workflow, turnover of research nurse staff requiring re‐introduction to clinic teams.	CNS leadership and active liaison role between academia and clinical practice, early and ongoing communication with stakeholders, integration of research tasks into existing clinic processes, visible appreciation and recognition for staff contributions, study topics perceived as clinically relevant and beneficial for Veterans.	Recommendations: Collaborative academic and clinical partnership enhances educational and professional environment for students and clinicians, promotes EBP and contributes to nursing scholarship.
Gonzalez‐de la Torre et al. (2023), Spain	To evaluate the attitudes, capacities and perceived barriers associated with research among nurses and midwives of the Canary Health Service (SCS).	Nurses and midwives (*N* = 512)	Quantitative descriptive	N/A	Attitudes towards Research and Development within Nursing Questionnaire (ATRDNQ‐e) instrument and the BARRIERS scale	Nursing profession does not require research‐based knowledge, nursing as a practice profession and does not need to include research, lack of nurses in clinical practice with a PhD, lack of time to implement ideas or do research.	Being part of a research team, linkage to university as a lecturer, membership of a society, having a nursing qualification, publication of poster or communication of research findings, organizational characteristics, graduate degree.	Recommendations: Provide nurses with time to engage in research.
Gullatte and Corwin (2018), United States	To describe the journey of the ‘One Emory Nursing’ initiative, which spans from bedside to bench.	Clinical nurses from Emory Healthcare's inpatient and ambulatory units; School of Nursing (SON) faculty; advanced practice nurses; nurse leaders; multi‐disciplinary collaborators (*N* = 35)	Qualitative case report	One Emory Nursing Initiative	N/A	Lack of protected time for clinical nurses to conduct research, administrative complexities for EHC nurses serving as PIs (adjunct faculty appointment requirement, post‐award fund management), technological barriers to shared platforms, challenges in joint communication and recognition of scholarly work across systems.	Strong leadership commitment from both SON and EHC, dedicated funding and positions for research facilitation and statistical support, cross‐representation on research councils, structured mentorship and small‐group coaching, financial support for dissemination activities, clinically relevant and mutually beneficial projects.	Outcomes: 12 collaborative studies between the faculty and clinical nurses, project presentations at conferences (including the Magnet conference), a Research Scholars Day, publications in peer‐reviewed. Nursing journals, research awards and successful grant funding.
Henshall et al. (2023), United Kingdom	To report on the development, implementation and evaluation of the first year of the NIHR 70@70 Senior Nurse and Midwife Research Leader Programme.	(*N* = 39) Senior nurses and midwives	Mixed methods	N/A	Online survey; written activity reports; thematic analysis of qualitative data; descriptive statistics of survey responses	Technical issues with NIHR Learn access; limited national collaboration opportunities; organizational constraints within NHS trusts; balancing programme with other commitments; varying seniority levels among participants; uneven geographic and specialty distribution.	Protected time for research; autonomy over objectives; strong programme communication; NIHR learn resources; national and regional networking; leadership support from chief nurses/executives; collaboration with higher education institutions; access to funding and training opportunities.	Outcomes: Programme's first year revealed that it successfully increased the research profile of nurses and midwives.
Higgins et al. (2010), International	To explore the experiences, challenges and benefits for nurses undertaking clinically based research in acute care and community settings.	N/A	Literature review	N/A	N/A	Perception that research is not real nursing work and a luxury, competing clinical demands and workload pressures, minimal managerial sponsorship for research involvement, limited familiarity with research language and processes, resource constraints (time, funding, infrastructure), disengagement and inconsistent participation from team members, scepticism from non‐research‐oriented nurses and other health professionals, research not clinically based, a good nurse viewed as clinically hands on, universities separate from the clinical setting, research ownership (conducted by nurses, not others like physicians), nurses feeling like research of others is imposed on them, culture that prioritizes practice, lack of understanding research language because it is not the culture, lack of research experience, prioritise short term goals.	Collaborative research teams sharing expertise, managerial and organizational sponsorship, research training, mentoring and formal education, opportunities for nurses to contribute to decision‐making in practice change, presentation of tangible benefits to patient outcomes to engage clinicians, journal clubs, education sessions, development of hospital based research positions, centres and councils, funded nursing fellowship, collaboration with academia and partnership with students, organizational characteristics (responsive administration, relational capital, hospital size), strong leaderships, positive environment, letting nurses take ownership of changing practice.	Recommendations: Valuing clinical research within organizations, ongoing funding for small and large scale projects, engaging services such as research assistants to keep projects moving forward, nurses collaborating with others who have experience. Outcomes: doing research builds capacity and enables nurses to think critically, an improve practice. Gives nurses a sense of achievement, increases engagement long‐term and improves benefits for patients.
Hwang et al. (2024), Korea	To identify and describe the sociocultural context of nursing research in a Korean tertiary hospital, focusing on factors that influence clinical nurses research activities, shared beliefs and perceptions.	Female RNs (*N* = 6) working in a medical surgical unit in a Korean tertiary hospital who had experience conducting nursing research	Qualitative ethnographic study	N/A	Participant observations and ethnographic interviews. Data was analysed using Spradley's ethnographic approach	Shift work and high workload fatigue, missed meetings, reduced research priority, uneven task distribution, low research competence, scheduling conflicts, limiting communication and training attendance, view that research is a separate entity from clinical role, challenge to juggle research and clinical role.	Hospital research support and education, funding, statistical consulting, submission portal for research questions, support for educational expenses, human resources to assist with research, clinically relevant topics from daily practice and higher engagement, flexible meetings (Zoom/KakaoTalk) and recordings for accessibility, positive team dynamics, master's degree and communication among team members.	Outcomes: Deepened understanding of need for change and creation of safer hospitals. Fostered engagement with clinical questions from clinical practice leading to practice changes and elevate the relevance of research to clinical practice. Increased leadership, expertise in the field, increased satisfaction, improved confidence, reinforced trust within the team, increased a sense of unity.
Jamerson and Vermeersch (2012), United States	To examine how nurse research facilitators (NRF) build research capacity among clinical nurses.	Nurses research facilitators (*N* = 34)	Quantitative descriptive	Nurse Research Facilitator	7‐item web‐based surveys on Survey Monkey. Total function count (TFC) was calculated for each participant to evaluate productivity	Part‐time roles or divided responsibilities, limited doctoral preparation, competing loyalties for faculty or dual‐role staff.	Full‐time employment within a clinical institution, doctoral preparation, strong administrative/CNO support, functional role model.	Outcomes: Doctorly prepared nurse research facilitators were the most productive in terms of functions performed, along with experience, % effort and relationship with the clinical institution.
Janerka et al. (2024), Australia	To describe the research capacity and culture (RCC) of nurses and midwives in two health services in Western Australia.	Nurses and midwives (*N* = 300)	Quantitative descriptive	N/A	Research capacity and culture tool	Lack of time, other work roles taking priority, lack of suitable backfill, staff shortages, lack of leadership, limited education/opportunity and culture not valuing research.	Desire to develop skills, increase job satisfaction, career advancement, addressing identified problems, team building and mentor availability.	Recommendations: Organizations should identify context‐specific barriers and enablers for research to plan and implement targeted capacity‐building and culture‐improving strategies.
Jeffs et al. (2013), Canada	To outline a Canadian hospitals multi‐pronged strategy to build nursing research capacity as a means to improve uptake of evidence in practice and ensure delivery of high‐quality patient care.	N/A	Mixed methods	Research capacity‐ building blueprint	Attendance tracking, number of research ideas submitted, number of posters, number of nurse‐driven studies generated, qualitative feedback from participants and leaders	Staffing shortages, limiting nurse release time, competing organizational priorities, fluctuating external funding availability and the need for sustained leadership engagement.	Senior leadership endorsement, dedicated director of nursing research, academic and professional partnerships, targeted funding streams, adaptable programme design, strong stakeholder engagement, alignment with organizational strategic priorities.	Recommendations: Articulate a vision for nursing research and a capacity‐building framework, employ a multi‐pronged approach, engage key stakeholders in planning and execution, have senior management endorse the value, leverage existing collaboration and seek opportunities to build research capacity and secure funding, (6) allow flexibility in trying new approaches.
Morrison et al. (2022), United Kingdom	To identify, evaluate and summarise current evidence in relation to the factors that influence the conducting of research by nurses in clinical practice.	N/A	Mixed methods (systematic review)	N/A	N/A	Competence, culture, low‐perceived theoretical knowledge, lack of knowledge, skills and research training, lack of time, lack of incentives, lack of mentorship with experienced nurses.	Research resources, protected time/backfilled time, mentorship from doctoral prepared nurses, access to training, collaborative approaches to learning, action research with a participatory approach, positive impacts to patient care, translatable findings into practice, dissemination of findings, characteristics of the nurse (curiosity, awareness, ownership, self‐confidence, eagerness to learn), feeling empowered, advancing professionally, increasing problem‐solving.	Outcomes: Identified four key themes influencing research activity—competence/culture, mentorship, resources and impact
Kelly et al. (2016), United States	To describe facilitators and hindrances to conducting registered nurse‐led research in U.S. hospitals.	RNs (*N* = 160) working in U.S. hospitals, from both Magnet‐designated and non‐Magnet facilities	Qualitative content analysis	N/A	Hospital‐based nursing research requirements and outcomes: open‐ended questions	Lack of time, absence of research mentors, insufficient training, inadequate infrastructure (onsite centres for research and grants, library services, online library resources and databases, biostatistical consultation, onsite IRB), lack of secretarial support, research assistance and coordinators, staff to support data entry, research design and grant writing, technology, informatics, lack of leadership support, minimal funding, unsupportive culture, competing priorities, low research relevance, nurse intimidation, lack of vision, view only physicians do research unaware of nurse‐led research, lack of experience, physicians and leaderships lack of understanding of nursing research, dominance in physician research, focus on patient care.	Research mentors (doctorally prepared, research consultants, advanced practice nurse), mentor from academic institution, one‐on‐one coaching (help with writing research question, develop study, IRB, monitor and evaluate project), strong leadership support, chief nursing officer who has commitment to research, institutional infrastructure, research training, relevance to practice, research committees, financial and tangible resources, dedicated time, higher nurse education, Magnet designation, integration into job roles, expectation that research is part of the job, residency programmes, hospital culture that values research, increasing knowledge, promoting professional development, culture of excellence and inquiry, start a nursing hospital‐based research programme, meeting Magnet requirements is a means to direct and facilitate nursing research, budget/financial support (admin assistant, consultations, computer software, research expenses, research development, travel, data entry, analysis).	Recommendations: Key to developing a nursing research programme is hiring research mentor who can provide leadership, guidance, support and training for staff nurses, hospital level committee, dedicated resources for training and conducting research, release time, create research as part of the culture, leadership with a vision.
Khair et al. (2012), United Kingdom	To determine barriers preventing haemophilia nurse specialists from engaging in research and to identify strategies to promote clinical research by haemophilia nurses in the UK.	Haemophilia nurse specialists and nurse consultants (*N* = 32)	Quantitative descriptive	N/A	Anonymous web‐based survey	Lack of dedicated/protected time, limited research knowledge and skills, difficulty identifying research areas, insufficient support from managers, low confidence and motivation, perception of lacking opportunities within current roles, lack of guidance on publishing.	Dedicated research time, managerial and peer support, mentorship from experienced nurse researchers, collaboration and teamwork, guidance on research and publication processes, participation in larger studies, online networking platforms (e.g., Haemnet) for collaboration and support.	Outcomes: All nurses felt they should engage in research.
Kress (2018), United States	To present practical strategies for overcoming barriers and encouraging clinical nurses to develop and use research skills in practice.	N/A	Discussion paper	N/A	N/A	Lack of research knowledge and skills, limited time due to clinical duties, lack of institutional or supervisory support, perception that research is not part of clinical nursing roles.	Formal and continuing education opportunities (courses, conferences, online modules), journal clubs and peer‐to‐peer discussion forums, use of institutional resources (medical librarians, nurse researchers), participation in shared governance/research councils, internal or external mentorship (including online mentoring networks), support through clinical ladder advancement and IRB training.	Outcomes: Clinical nurse participation in research is vital to providing high quality care to patients. Professional development educators can help clinical nurses overcome barriers.
Krewulak et al. (2024), Canada	To describe ICU care team members experiences with ongoing ICU research, identify perceived barriers and enablers to their participation and apply the Capability, Opportunity, Motivation, Behaviour (COM‐B) framework to enhance research engagement.	ICU care team members: registered nurses, allied health professionals, attending/fellow physicians, nurse practitioners (*N* = 172)	Quantitative descriptive	N/A	Online qualtrics survey developed for this study	Low confidence in discussing research with patients/families (especially among RNs, allied health, clerks), limited perceived role in research facilitation, time constraints, low awareness of ongoing studies, especially where research offices are not onsite.	Proximity to research offices, integration of research discussions into multi‐disciplinary rounds, discipline‐specific research champions, recognition through authorship or acknowledgment, sharing results and highlighting clinical impact, structured communication, mentorship from research teams.	Recommendations: To increase ICU research, healthcare professionals must be educated with knowledge and skills, given opportunities (resources and support) and motivated.
Landeen et al. (2017), United States	Describes the delivery and evaluation of a hope research community of practice (HRCoP) approach to facilitate research expertise in a group of advanced practice nurses in one hospital.	Masters‐prepared nurses (three advanced practice nurses, four in leadership roles) from a large multi‐site teaching hospital in Southcentral Ontario (*N* = 5)	Qualitative descriptive	Hope Research Community of Practice (HRCoP)	Semi structured qualitative individual interviews focusing on confidence in conducting hope related research, participant observation	Competing clinical and personal demands limiting protected research time, challenges with research ethics applications and adapting to new online systems, difficulty balancing high‐quality research aims with learning needs and time limits, small group size magnifying impact of dropouts, limited social interaction outside scheduled sessions.	Strong leadership and administrative endorsement, structured, supportive learning environment with expert facilitation, common unifying theme of hope, fostering shared language and interest, access to hospital/university research resources (e.g., biostatistics, REDCap, librarians), collaboration across departments and professional roles, encouragement to normalise research challenges and share solutions.	Combining academic and clinical nursing resources helps practicing nurses and health‐care organizations promote nursing research. Operational pressures that prevent practicing nurses from engaging in research must be addressed.
Latimer and Kimbell (2010), United States	To describe the Nurse Research Fellowship that was designed to educate nurses on the research process and enable nurses to lead research projects.	Nurses (*N* = 11) accepted into the fellowship	Mixed methods	Nursing research fellowship	N/A	Limited research knowledge and experience, time constraints, lack of support for nursing led research (preparing protocols and IRB submissions) and limited resources.	Structured programme, mentorship and collaboration, leadership support, culture of inquiry, collaboration with hospital departments.	Outcomes: All nurses were able to develop a research proposal. Barriers such as time and money were addressed in the fellowship. Increased nursing research projects and knowledge. Requires mentorship for novice researchers. Retention was an issue. Time and money were not provided for implementation, which is a limitation. Magnet status was attained.
Lehmkuhl et al. (2020), United Kingdom	Explore critical care nurses experience of research participation during a 1 year recovery programme for intensive care survivors.	Trained ICU nurses in Denmark (*N* = 13)	Qualitative descriptive study	N/A	Focus groups, interviews	Limited research skills, time constraints, competing clinical demands, navigating institutional processes and lack of awareness of research opportunities.	Collaboration with experienced researchers, mentorship, organizational support, professional curiosity and recognition of research value to patient care.	Outcomes: Nurses found participation in a research project positive, enhancing their personal growth, research competencies in the practice setting, stimulated new knowledge, broadened their perspectives and increased critical thinking. Recommendations: Nursing leaders and organizational managers should prioritise nurse involvement in clinical research to increase competency and support nurses doing research to help further their professionalism and clinical leadership.
Lieschke et al. (2022), United Kingdom	Identify existing research capacity/capability and requirements necessary to promote, support and enable an embedded sustainable nursing and midwifery research culture within LHD.	Phase 1 survey: nurses (*N* = 721) and midwives (*N* = 95) Phase 2 focus groups: senior clinicians/educators/managers (*N* = 33)	Mixed methods (case series)	N/A	Semi‐structured interviews; thematic analysis identified perceptions of research capacity, culture and influencing factors	Lack of time, limited structural/organizational support and resources, competing KPIs, managers valuing research less than clinicians, rural sites with less support.	Clear patient outcome relevance, being engaged as key contributors, recognition/incentives, research conducive culture, partnerships, mentorship/scholarship programmes, links to expertise (e.g., statisticians) and translational/implementation focus.	Recommendations: There needs to be preparation on the individual, unit and organizational level, partnerships, mentorships and scholarships and links for research expertise and support for translational research to occur.
Loke et al. (2014), United Kingdom	Explore clinical nurses' perceptions of their research knowledge/experience and identify gaps in education and organizational support for conducting research.	Clinical nurses (*N* = 146) who attended 4 research seminars in Singapore	Mixed‐methods (exploratory descriptive design)	N/A	A questionnaire developed for the study with open and closed ended questions	Time constraint, research should be done on own time, lack of organizational support, skewed resource distribution, limited knowledge of external funding, managerial ambivalence, research viewed as extra work without protected time, physicians have research opportunities, resources and privilege.	Shift education towards hands‐on research experience, provide protected time and financial support, strengthen transparency and clear guidelines between researchers, institutions and patients involved, supportive managers/doctors and broaden awareness of available opportunities.	Outcomes: Nurses voiced enthusiasm about engaging in research but encountered frustrations with organizational culture and infrastructure. A strong emphasis on educational training. Where educational opportunities exist, all nurses need to be made aware.
Martin (2017), Sweden	To explore attitudes towards research among a subgroup of Swedish nurses specializing in ophthalmic care and to compare the results with the findings of a similar study from 2001.	Invited ophthalmic Swedish nurses (*N* = 250)	Quantitative descriptive	N/A	Web based questionnaire	Lack of time, older age/imminent retirement, heavy workload and patient‐care backlogs, limited managerial/collegial support, perception that research is for physicians, uncertainty about how to start/fund projects, frustrating prior experiences, complex research language.	Protected research time, stronger managerial encouragement and resources, accessible research training/mentorship (including non‐physician lecturers), clearer, user‐friendly research communication, workplace access to research literature, opportunities to engage with conferences/students to stimulate development.	Outcomes: Little has changed in the past decade regarding nurses' attitudes towards research. The assumption that specialist nurses would be more interested in research was not supported.
Matikainen (2017), Australia	Explore the legitimacy and agency of new graduate mental health nurses to participate in research activities as a regular part of their professional nursing role.	New graduate nurses	Discussion paper	N/A	N/A	Personal: low confidence, limited research skills/knowledge, difficult transition to practice, negative ward attitudes, power differentials. Organizational: rotation stress, unclear role expectations (researcher vs. user), limited release from ward duties, staffing coverage issues. Time/workload: busyness, paperwork, non‐nursing tasks, lack of paid time for research.	Leadership & structures: clinical supervision/mentorship, research units (e.g., MHNRPDU); manager‐approved release time to legitimise participation. Learning culture: journal clubs, research updates at meetings, access to journals/computers, dedicated crossover time (e.g., night‐shift overlap) for education/research. Career development: identification/support of interested new grads, translational projects, supportive peers.	Recommendations: All nurse should be involved in research at some point, newly graduate nurses are well positioned to participate in the research process, research engagement benefits nurses, health services and improves clinical care, strategies to improve new graduate engagement include: supervision, senior management support and promoting a learning community, organizational support for staff participation in research builds capacity, promotes job satisfaction, employee loyalty and increases staff retention.
Mazzella Ebstein et al. (2020), United States	To describe the development, implementation and outcomes of a model for building research capacity among oncology nurses.	RNs employed at Memorial Sloan Kettering Cancer Centre (*N* = 18)	Mixed‐methods	Nursing Research Fellowship	Self‐evaluations before and after completion of the fellowship programme, evaluation surveys, observation of programme outcomes such as presentations and projects initiated	Competing clinical demands and workload pressures, limited prior research experience among staff, scheduling conflicts for education and mentorship sessions.	Protected time for research activities, leadership endorsement and visibility of the programme, structured mentorship from experienced nurse researchers, accessible education tailored to direct care nurse's needs, supportive infrastructure, including research councils and recognition of participation.	Recommendations: Utilizing eLearning, videos and teleconferencing can expand research fellowship programmes beyond the classroom. Requirements include: Transparency in application process, availability of nurses to attend classes and inclusion of nurse‐scientists.
McKee et al. (2017), Ireland	To describe the implementation of an intervention to increase research participation in advanced clinical nursing roles and evaluate its effectiveness.	Nurses with advance nursing roles (*N* = 72)	Quantitative descriptive	Intervention to increase clinical nurse research participation	One questionnaire (self‐reported research skill utilisation and development questionnaire) and quantitative audit information	Time to do research, attend meetings, lack of protected time, not knowing research takes time (cleaning data, IRB), lack of awareness research is part of their work, voluntary participation of the academic nurse researcher and clinical nurse researchers, mentors not holding expertise on the topic, lack of clarity of roles, ethical issues, time consuming nature of research, lack of access to resources (computer programmes), fear research will not be published.	Teamwork, support, collaboration (merging expertise, setting deadlines, goal setting).	Outcomes: 12 nurses participated in the intervention, all had advanced degrees. 15 projects are active. Nurses published papers in peer‐review journals, increased skill development and research skills (methodology, writing of manuscripts, formulating a research question). The intervention offered nurses stimulus, motivation, opportunity and support to publish findings. Improved clinical outcomes, increased observation of their clinical practice and improved patient care.
McLaughlin et al. (2013), United States	To describe the programme requirements and scholarly outcomes for RN led research in US hospitals.	Hospitals (*N* = 160) both magnet and nonmagnet	Quantitative descriptive (national survey)	N/A	Hospital‐Based Nursing Research Requirements and Outcome survey	Competing clinical workload and limited time, variable research experience and skills among nurses, inconsistent availability of funding or resources.	Magnet programmes emphasis on research and EBP, presence of dedicated research staff and committees, organizational policies supporting research processes, funding and support for dissemination activities.	Outcomes: The top facilitator was a research mentor and the top hindrance was lack of a research mentor. Thirty‐three hospitals (21%) had research education classes. The majority of hospitals had education contact hours and paid nurses to attend the course. Twenty present provided nursing research fellowships/internships. Sixty five percent had research council/committees, 82.5% had RN‐led research studies.
Montgomery et al. (2021), United States	To describe benefits and challenges of incorporating Advanced Practice Nurses (APNs) in research and outline opportunities to build capacity for nurse‐led research.	Children's hospitals (*N* = 5); research teams included advanced practice nurses (APNs) (*N* = 36), RNs (*N* = 8), physicians (*N* = 9), research staff (*N* = 10)	Case study	N/A	Author's description using field notes (monthly meetings, sit initiation visits, team meetings, writing retreat)	Lack of protected time, constrained regulatory/coordination support, inadequate financial infrastructure for APN‐led research, variable leadership support and recognition.	Structured mentorship, clear role delineation, foundational training, multi‐layer support model, leveraging grant funds to buy out time, opportunities for continuing education and dissemination.	Outcomes: APNs have a professional responsibility to engage in research to improve quality of care. Organizational infrastructure is a critical factor to promote hospital nursing research. Recommendations: mentor opportunities, leaders and APNs should consider evaluation of benefits and challenges of engaging in research, APNs and leadership should conduct comprehensive assessment of research infrastructure.
Morrison et al. (2022), United Kingdom	To identify and synthesise factors influencing research activity among nurses, midwives and allied health professionals (NMAHPs) using a mixed methods systematic review.	Studies including nurses, midwives and allied health professionals at various career stages (*N* = 37)	Mixed methods (systematic review)	N/A	N/A	Lack of time, insufficient skills, inadequate funding, absence of organizational support, competing clinical priorities, low self‐perceived theoretical knowledge and practical research competencies, lack of acceptance from colleagues, lack of and training (writing grant applications and protocols), access to mentors, low access to research resources.	Access to training/mentorship, protected research time, supportive leadership, strong research culture, interdisciplinary collaboration, external partnerships, trigger events, nurse characteristics (curiosity, awareness, ownership, self‐confidence, eagerness to learn), nurses feeling empowered, being part of the bigger picture, advancing professionally, collaboration, passion to improve care.	Outcomes: Despite lack of training, nurses are motivated to engage in research. Recommendations: clinical nurse leaders and managers should increase efforts to support nurses to address extrinsic barriers to conducting research.
Nixon et al. (2013), United Kingdom	To engage nurses and midwives in a trust‐wide research project.	Nurses and midwives (*N* = 50)	Descriptive practice report	Research Workshop	Survey questionnaire	Lack of research knowledge and skills among clinical nurses, lack of time, staffing issues, lack of support, lack of awareness, lack of resources, lack of expertise, competing clinical workloads, insufficient protected time for research.	Dedicated nursing research office and infrastructure, experienced mentors, visible organizational commitment, structured support for ethics, project design, analysis and dissemination, culture shift promoting the value of nursing research.	N/A
Nowlin et al. (2021), United States	To describe the knowledge, attitudes and practices of research among RNs at an academic healthcare system.	RNs (*N* = 99)	Quantitative descriptive	N/A	Nurses' Knowledge, Attitudes and Practices of Research Survey	Lack of skill sets and knowledge required to put together research proposals and evaluations, nurses rated their knowledge and skills in critical areas as low, small number of courses covering research methods, lack of time and institutional support.	Mentorship structures to further develop translational research skills in the practice setting, expert researchers and novice researcher partnership, establishing cost‐neutral or cost‐saving programmes.	Outcomes: As nurses have increased their level of education, their ability to engage in research increases.
O'Brien et al. (2022), United Kingdom	To explore the experiences of a nursing team who implemented an international nurse‐led clinical trial in practice and understand the facilitators to their involvement.	Nurses employed at the cancer centre (*N* = 18)	Qualitative descriptive	N/A	Focus groups with a semi structured topic guide	Lack of time and heavy workload, lack of research experience and training, insufficient skills or confidence, limited organizational and managerial support, research as being predominantly medically led.	Leadership and organizational support, mentoring and academic collaboration, dedicated research positions, training and education opportunities, recognition of research in professional development, consistent and transparent communication about research/study, researcher understanding the context (nurses had trust in the research team), perceived benefits to clinical practice was motivating, research leading to enhanced education and conferences, feeling of ownership, recognition they were improving practice, communication within the research team, helps for job promotions.	Outcomes: Nurses felt they have potential to make positive changes when engaging in research. Nurses learned research concepts by participating in the study.
Patton et al. (2020), United States	Describe strategies to engage direct care nurses in multi‐site research (PEDSS study) and address barriers to Magnet related nursing research.	Magnet‐designated hospitals (*N* = 16); RNs (*N* = 86)	Case study	N/A	Semi‐structured interview guide; audio‐recorded, transcribed verbatim	Lack of time, limited fiscal resources, minimal infrastructure, need for IRB coordination, communication challenges, variable leadership support.	Leadership engagement and support (CNO, managers), research mentors (PhD‐prepared nurses), centralized toolkit, site investigator and study personnel training, communication with study personnel and stakeholders (monthly calls and annual meetings), study workflow integration, recognition via clinical ladder, dissemination support.	Nursing practice is improved as nurses engage in research and knowledge generation. Nurses' engagement in research provides them with an opportunity to learn and develop research skills and foster relationships between experienced and novice researchers.
Pepper et al. (2024), United Kingdom	Aimed to assess the research involvement and support needs of Admiral Nurses and identity factors affecting their participation in research.	Admiral nurses (*N* = 381) working in any care setting across the UK	Mixed methods	N/A	Anonymous online survey including both quantitative and qualitative questions	Lack of time to read or carry out research, lack of confidence in carrying out research, lack of support from others, lack of motivation to participate in research.	Support from their peers in the form of a network of practice to become more research minded, coaching or mentorship, workshops on writing or performing research.	Outcomes: Actual participation of specialist dementia nurses is limited due to significant barriers such as time constraints, lack of support and low confidence.
Pintz et al. (2018), United States	Describe research characteristics, infrastructure and productivity across Magnet‐designated hospitals.	Magnet‐designated hospitals (*N* = 181); nursing leadership respondents	Quantitative descriptive	N/A	Online Hospital‐Based Nursing Research Characteristic Questionnaire	Limited dedicated nurse scientist roles, insufficient funding, lack of protected time, variation in infrastructure, lack of biostatistician support, quantitative software, qualitative software, grant writing assistance, free external IRB services, lack of physician support.	Magnet requirements, research councils, mentorship programmes, academic partnerships, leadership support, designated nurse scientists, encouragement to pursue higher education, library resources and access to databases, on‐site library resources including medical librarian, on‐site IRB, multi‐disciplinary collaboration, reimbursement for graduate tuition, institutional research culture, paid time, research education, research committees, financial assistance and resources, documentation of scholarly outcomes.	Outcomes: Hospitals with nursing research departments, 10 RN‐led studies in the past year had better ratings on hospital infrastructure, research culture and more published research. There was a consistent lack of internal research funding.
Ramon et al. (2022), Spain	Identify motivation levels and barriers to research among nursing professionals in southeast Spain.	Nursing professionals (*N* = 91)	Quantitative descriptive study	N/A	Questionnaire	Time constraints, lack of research training, scarce institutional support, few incentives and weak integration of research into roles.	Access to training, managerial/institutional support, incentives and recognition, integration of research into daily practice, engagement of younger/educated professionals.	Outcomes: Although nurses have a positive attitude towards performing research, the percentage of nursing professionals that perform research is low. Recommendations: Health managers should provide adequate resources to develop research skills among nurses.
Robichaud‐Ekstrand (2016), Canada	Examined clinical nurses views on nursing research and identified some contributing factors to the research‐practice gap.	RNs (*N* = 1081) from New Brunswick, Canada	Quantitative descriptive	N/A	Questionnaire to evaluate participants' views on nursing research	Lack of time, insufficient knowledge/training, resource constraints, limited organizational support, lack of resources and awareness of, difficulty understanding research and statistics, lack of mentorship, insufficient hospital‐academic partnership, lack of nurse participation, lack of confidence in research competencies, lack of decision makers' involvement in research, lack of competencies using technology, lack of funding.	Managerial support, access to research, favourable attitudes towards EBP and education opportunities.	Outcomes: Nurses viewed research favourably, however, engagement was sparse. Younger nurses were more eager to engage in research activities and more competent using technology. Higher education positively influenced nurses' views of research. More years of experience was related to increased research experience. Recommendations: research supervision, utilizing participation in collaborative clinical‐academic research networks, involve patients, community and policy makers to improve project relevance.
Scala et al. (2019), United States	To gain an understanding of what motivates clinical nurses to be interested and/or engaged in research; describe motivators; identify common characteristics (profile).	Clinical nurses (*N* = 34) who identified as having research interest across 7 sites	Qualitative interpretive description	N/A	1:1 interviews	Lack of awareness and/or opportunities to engage, constraints of time/resources, system‐level strategies often miss the individual nurse level.	Feeling empowered to make a difference, professional obligation, quality care/helping people, learning new knowledge, social engagement/team work, professional advancement, networking, increased satisfaction, opportunities such as fellowships, interest in the topic, increase credibility of the nursing profession, career advancement, having a future impact, opportunities, incentives.	Outcomes: Being part of research empowered them to make a difference, legitimise the profession and was viewed as a professional obligation. Research gave nurses a voice to improve health care and make system level impacts. Research connects nurses to the larger picture and enables nurses to utilise the full extent of their degree. However, there were limited opportunities. Recommendations: organizations and leadership need to support nurses in research engagement, helping them acquire knowledge and new behaviours. It is important to tap into motivators such as professional growth, career satisfaction, growth of the discipline and credibility.
Scala et al. (2016), United States	To review the literature for best practices for engaging clinical nurses in nursing research.	(*N* = 19) articles	Integrative review	N/A	Johns Hopkins Nursing Evidence Based Practice Model	Lack of time, workload pressures, limited research knowledge, insufficient organizational support and minimal access to mentorship.	Administrative and leadership support (CNO AND CEO), mentorship, research infrastructure, recognition of contributions, development of a research office or institute lead by doctorally prepared nurses, alignment with clinical and organizational priorities, relevant research, encourage nurses to gain advanced education, connecting research with ongoing EBP activities, involving clinical nurses in IRB process, creating nursing councils, onsite libraries, biostatistician, online access to articles, support with data management, access to grant funding, have a budget dedicated to paid and protected time, speaker honoria, small grant competitions, relevant topics to clinical care, ask nurse for research topic, engage nurses in all aspects of the research process, research mentor makes themselves visible, established education and training programmes, research fellowship, guidance writing funding proposals and completing IRB, academic‐hospital partnerships, research champion.	N/A
Scarsini et al. (2022), Italy	Identify barriers and enablers of conducting nursing research as perceived by Italian nurses.	(*N* = 8) studies	Systematic review	N/A	N/A	Poor English language, limited technology/library availability & accessibility, understaffing and lack of time, nursing culture characteristics, lack of organizational support, lack of nursing leadership support, scarce funding, bureaucratic ethics‐committee processes.	Reading nursing journals, support from expert research teams, university hospital partnerships.	Many obstacles decrease Italian nurses from engaging in research, including: individual, organizational and system‐wide levels.
Shepherd et al. (2022), United Kingdom	To develop initiatives to facilitate clinical research opportunities and bridge the gap between clinical care and research.	Organizational initiatives at Royal Devon and Exeter NHS Foundation Trust described, nurses, midwives, allied health professionals	Discussion paper	Strategies to increase staff awareness and engagement in clinical research	Participation rates in programme activities, number of projects initiated, qualitative feedback from participants, programme sustainability indicators	Competing clinical priorities, limited time for staff to engage, lack of awareness of research opportunities, traditional separation of research and clinical care.	Visible leadership roles (e.g., research nurse leaders), institutional strategies, designated research champions, integration of research into governance structures, awareness campaigns that normalized research as everyday practice.	Outcomes: After 6 months participation in the fellowship nurses increased activity in applying for research, writing protocols, using qualitative research methods, publishing, writing a research report, analysis and interpreting results, using quantitative methods, finding relevant literature, generating research ideas.
Shepherd and Quinn (2024), United Kingdom	To implement and evaluate a strategic plan for research within a Southwest Foundation Trust, aligning with the national Chief Nursing Officer (CNO) research strategy.	NMAHP staff across a Foundation Trust	Case report	Research Strategy	Self‐assessments of research experience.	COVID staffing disruptions, limited ward‐level integration, need for long‐term cultural change.	Executive oversight and leadership, mentorship and role‐modelling, funding (NIHR, Trust), University and NIHR network partnerships, peer support via Clinical Academic Network.	Outcomes: 17 individuals completed the programme with positive evaluations, presented work at international research conferences, many considering research careers. Nurses collaborated, received peer support and mentoring, developed proposals and applied for funding.
Siedlecki and Albert (2017), United Kingdom	To develop a theoretical understanding of factors influencing clinical nurses decisions to conduct research.	RNs (*N* = 26) who worked in a hospital or ambulatory setting and had participated as principle investigator on at least one completed clinical nursing research study	Qualitative grounded theory	N/A	Individual interviews	Limited research exposure in education, lack of confidence and belief that research is only for PhDs. Time constraints (especially for bedside nurses), challenges with dissemination due to poor writing preparation.	Clinical triggers prompting inquiry, access to mentors and role models, supportive organizational culture (library, statisticians, funding, recognition), peer involvement and manager encouragement, passion and personal commitment to patient care improvement.	Outcome: Clinical nurses can engage in research when given access to mentors and are in a research supportive environment. This is true even if they lack time, funding and knowledge.
Snelgrove and James (2011), United Kingdom	Explore graduate nurses' perceptions of the local nursing R&D culture and identify barriers/facilitators to research participation within one health trust.	Graduate nurses (*N* = 20)	Qualitative descriptive	N/A	Focus group interviews	Insufficient/protected time, heavy workload and family and work demands, complex/onerous ethics and governance processes, lack of education, unsupportive or research‐neutral teams/managers, perceived medical dominance, apathy among non‐graduates, theory‐practice gap and loss of skills/confidence over time, mentorship.	Research‐friendly culture (supportive managers/teams), mentorship (including interprofessional allies), protected time, projects tightly linked to service priorities and everyday practice (e.g., action research), higher‐education modules that demystify methods and focus on practical applicability, institutional infrastructure (R&D groups, governance pathways) when accessible, research projects that fit within daily work flows, research friendly teams.	Outcomes: Graduate nurses hold positive views towards research. They are most motivated when research is relevant to nursing practice, fit with institutional strategies and positively impacted patient care.
Stutzman et al. (2016), United States	To evaluate the effectiveness of a Neuroscience Nursing Research Center (NNRC) fellowship model in engaging bedside nurses as primary investigators in clinical research.	RNs (*N* = 20) enrolled between 2013 and 2016 and 150+ additional nurses that were trained or reached through workshops, boot camps and consultations	Descriptive programme evaluation	Neuroscience Nursing Research Center (NNRC) and Fellowship	Programme outputs included number and type of research projects, publications, presentations and perceived professional growth; qualitative feedback from participants; alignment with Magnet criteria	Initial resistance to recognizing nurses as primary investigators, few nurses on IRB committees, limited time/resources, difficulty scaling to multi‐centre research.	Strong administrative support, structured fellowship with mentorship, tailored infrastructure (consultation, boot camps, writing support, grants), visible programme outcomes recognized in Magnet designation and conferences.	Outcomes: 20 nurse research fellows, each as PI on IRB approved study. Five research grants, over $25,000 to support their research. Forty seven projects, fellows have enrolled over 256 nurses and 683 patients in studies. Over 150 nurses received research training. Over 20 abstracts or posters, 11 manuscripts submitted.
Turjanica et al. (2013), United States	To describe the Nursing Research Idea Fair as a strategy to foster research interest, reduce fear about research and increase direct care nurses engagement in research activities.	Attendees at Nursing Research Fair in Northeast Ohio; 200–500 attendees per fair	Programme description and evaluation	Dedicated Neuroscience Nursing Research Fair	N/A	Lack of time, research knowledge and support; initial fear and anxiety around research.	Fun, non‐intimidating environment (themes, games, prizes, food), support from the Nursing Research Council (NRC) and administration, mentorship and collaboration with experienced researchers, incentives (e.g., magnet conference sponsorship, gift baskets), public recognition through local and national media coverage.	Outcomes: The fair was an opportunity to educate nurses about research and increase their involvement. It used fun and inviting methods to have them engage in research. 200–500 nurses have attended. Twelve posters per year and 27 research ideas submitted. The quality of the ideas has increased. Up to 8 studies per year have resulted from the fair. Nurses now see themselves as being able to conduct research and practice changes have been witnessed.
Twelvetree et al. (2019), United Kingdom	To describe an approach to developing sustainable nursing and allied health professional (NMAHP) research capacity by supporting postdoctoral clinicians to remain in practice while developing research skills.	Postdoctoral NMAHPs (nurses, physiotherapists, etc.) at Central Manchester University Hospitals NHS Foundation Trust	Programme description	Practice base approach to support post‐doctoral clinical academics	Action learning framework	Limited time and protected research opportunities, competing clinical workloads and patient caseloads, high competition for fellowships and academic posts, lack of recognition of research as part of clinical consultant roles.	Strategic buy‐in from senior managers and academic leadership, Action learning framework supporting collaboration and reflection, dedicated research time negotiated with line managers, mentorship, team reflection and multi‐professional collaboration.	Outcomes: Clinical academics now attain experience developing studies, receive support and continue in practice. Individual research abilities increased and capacity was built within the organization. Organization research priorities were identified and patient care improved. Professional expertise, drive and strategic buy‐in were needed for change. Recommendations: activities should not be solely to increase research, but to create a research culture within the organization that is sustainable.
Walden et al. (2015), United States	To engage staff nurses in research in an informative and fun way by examining their preference between sugar‐free and regular chocolate chip cookies, while simultaneously teaching them about the research process.	Paediatric and obstetric staff nurses at Texas Children's Hospital (*N* = 300)	Mixed methods	Great American Cookie Experiment (GACE)	The cookie assessment tool	Initial fear and intimidation regarding research, limited prior experience in research methods, demands of clinical workload limiting time for research.	Fun, hands‐on experiential learning (cookies, blind tasting), structured mentorship via research scholars programme, support from nursing research council and faculty, integration into Nursing Grand Rounds for dissemination, use of conceptual frameworks (Benner, Wright) to guide novice‐to‐expert progression.	Recommendations: Practice environments that engage nurses generation and dissemination of research are needed to maintain high standards of professional practice and ensure high quality nursing care. This can be done by developing a culture of research and EBP. Conceptual frameworks and structured educational programmes can be used in healthcare organizations to facilitate a culture of inquiry.
Walsh et al. (2023), Australia	To understand barriers and enablers experienced by nurse/midwife clinician researchers in Australian health systems.	Registered nurses or midwives (*N* = 15)	Qualitative descriptive study	N/A	Semi‐structured interviews	Undervaluation by leaders/peers, unclear career pathway, insufficient capacity and lack of confidence, personal sacrifice of time, insufficient funding, limited motivation/mentorship, weak leadership support.	Protected research time, supportive leadership that values nursing research, capacity‐building initiatives, (proposed) formal recognition and mentoring structures.	Outcomes: Despite the barriers, clinicians had a passion and tenacity to do research, motivated by seeing the impact it has on practice. Recommendations: healthcare organizations and universities are responsible for creating a culture that supports clinical research. Nurse/midwife clinical researchers should be recognized and valued; formal mentoring programmes are required; protected time and payment for the work is required; structured pathways for sustainable research clinician career progression need to be developed.
Weitzel and Robinson (2011), United States	To promote staff nurse awareness of research activities and encourage research engagement through a week‐long celebration at a large Midwestern teaching hospital.	Staff nurses from 11 nursing units (*N* = 288)	Qualitative descriptive (programme evaluation)	Nursing Research Week	N/A	Limited prior research education among staff nurses, lack of time and perceived authority to implement practice change, insufficient communication/advertising left some unaware of activities.	Diverse, engaging activities tailored to varying research knowledge levels, organizational and managerial support, interactive, participatory learning (cookie experiment, poster sessions), collaboration with local nursing schools and Sigma Theta Tau chapter, dissemination of results through newsletters and hospital library displays.	Recommendations: When planning events, have sessions geared to those with basic understanding and involve other disciplines.
Westlake et al. (2024), United States	To describe heart failure (HF) nurses research interest, involvement, confidence, facilitators/motivators and barriers to leading or collaborating in research studies.	HF nurses in the United States (*N* = 145) working in various settings	Quantitative descriptive	N/A	Adaptec instrument from the National Association of Clinical Nurse Specialist Organization	Lack of authority to initiate research or seek funding, limited support for data analysis and proposal writing, insufficient personnel/resources, knowledge gaps in protocols and research analysis, research not included in annual performance evaluations.	Control of schedule/work time, participation on research councils or committees, academic appointments, perception that research presentations improve HF care, organizational/mentorship support opportunities.	Recommendations: Nurses of all levels need assistance to participate in research and receive education on research and EBP. Nursing research is needed to ensure safe equitable care is provided to patients.
Williams et al. (2024), Australia	To explore the perspectives of clinician nurses working as research assistants in a nurse‐led research project.	Clinician nurses (*N* = 8); all worked as research assistants in a nurse‐led project at a tertiary hospital in Western Australia	Qualitative descriptive	N/A	Semi‐structured interviews	Clinical workload and patient acuity limiting recruitment, COVID‐19 demands reducing time/energy, lack of accessible research opportunities, training and mentors, confusion about nurse role vs. researcher role.	Strong motivation to improve patient care, relevance to specialty practice, clear role definition, training, supportive research team presence and flexibility in rostering.	Recommendations: Supporting clinicians in nurse‐led research fosters their learning of research. Appropriate training and mentorship are required. This can improve job satisfaction and translation of research to practice, improving clinical outcomes. Leaders must support these activities by providing protected time, mentorship and internship programmes.
Woith et al. (2014), United States	To examine unexpected barriers to the conduct of hospital research during a study of nurses' activation of rapid response teams.	Hospital nurses in a faith‐based community hospital	Qualitative reflective study	NA	Semi structured interviews	Lack of time and difficulty to schedule, lack of around the clock availability of researchers to engage with nursing staff, lack of interest in leaving patients to engage in research.	Incentives for participating in research, advanced degrees or learning opportunities in regards to research, relevance of the research, belief in the importance of research, flexibility.	Conclusion: Bedside nurses are experts in providing safe, excellent care and can provide a wealth of information. Consequently, their participation in research is vital so researchers need to be proficient at conducting hospital research. This entails having a realistic understanding of potential barriers.

## 4 Results

A total of 11,051 records were identified through database searching on April 28, 2025. After duplicates were removed, 5544 articles were screened by title and abstract. Of these, 830 full‐text articles were assessed for inclusion, resulting in 286 articles included in the larger scoping review. During data extraction, 79 of the 286 were identified as articles reporting factors specific to nurses' engagement in research and are reported in this paper. Articles reporting nurses' engagement in EBP and in EBP combined with research will be reported in future manuscripts.

### 4.1 Characteristics of Included Articles

A total of 79 articles were included in this review. There were a variety of methods, including: quantitative descriptive (*n* = 26), qualitative (*n* = 21), mixed methods (*n* = 14) and reviews (*n* = 3). Other methods included: concept analysis (*n* = 2), case study (*n* = 5), discussion paper (*n* = 3), descriptive practice report (*n* = 1), programme description (*n* = 2) and descriptive programme evaluation (*n* = 2). No articles were randomized controlled trials. Dates ranged from 2010 to 2024 and all articles were published in English. Countries where the articles were conducted included: United States (*n* = 28), United Kingdom (*n* = 24), Australia (*n* = 8), Denmark (*n* = 3), Canada (*n* = 3), Sweden (*n* = 3), Norway (*n* = 2), Italy (*n* = 2), Spain (*n* = 2), Korea (*n* = 1), Ireland (*n* = 1) and International (*n* = 2). See Figure 2 for a representation of the distribution of countries. See Table 1 for a summary of included articles.

**FIGURE 2:**
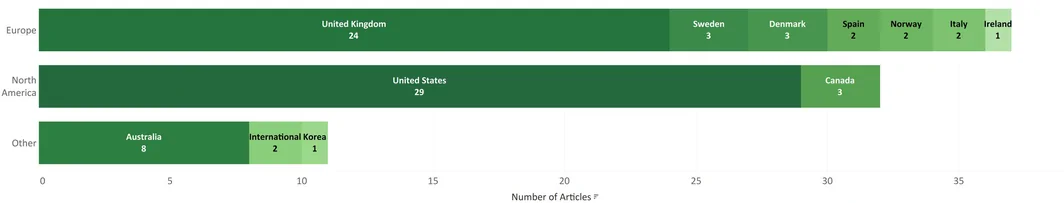
Representation of the distribution of countries. [Colour figure can be viewed at wileyonlinelibrary.com]

### 4.2 Barriers

During data analysis, it was evident that factors influencing nurses' engagement in research were either barriers that prevented nurses' engagement or facilitators that enabled nurses' engagement. Barriers to nurses' engagement in clinical research included: (a) insufficient time, (b) inadequate research experience, training and mentorship, (c) lack of motivation, (d) insufficient leadership support and (e) an unsupportive research culture within the organization. See Figure 3 for a representation of the main barriers and their corresponding references.

**FIGURE 3:**
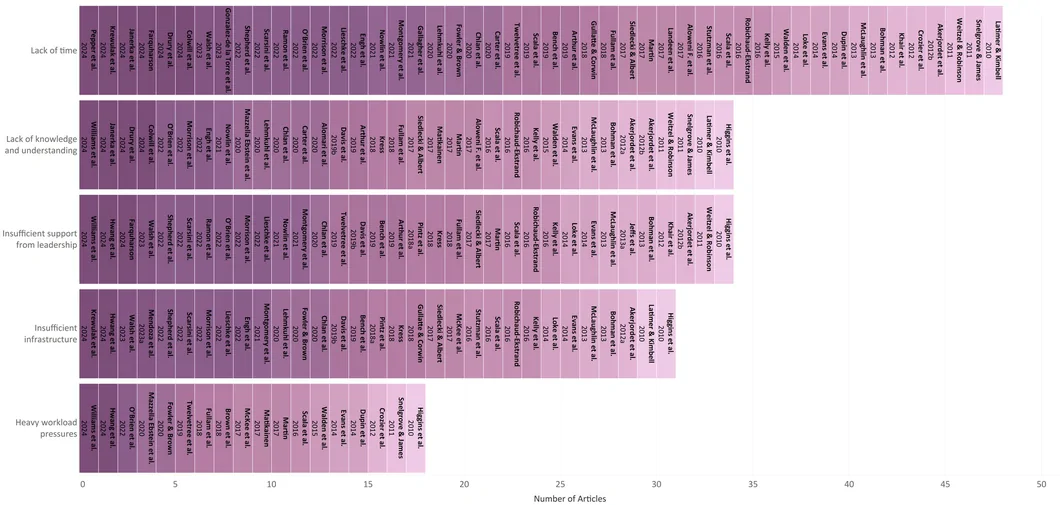
Representation of the main barriers to nurses' engagement in research and the corresponding references. [Colour figure can be viewed at wileyonlinelibrary.com]

#### 4.2.1 Insufficient Protection of Research Time Amid Competing Clinical Demands

The most frequently reported barrier to nurses' ability to engage in research was a lack of time, reported in 47 articles. Reasons for a lack of time included: nurses were not provided with designated and protected time away from clinical duties (Akerjordet et al. 2012a; Arthur et al. 2019; Davis et al. 2019; Higgins et al. 2010; McKee et al. 2017; Mendoza et al. 2023; Montgomery et al. 2021; Pintz et al. 2018; Snelgrove and James 2011; Twelvetree et al. 2019), shift patterns that did not allow nurses to attend meetings or participate in research activities (Bench et al. 2019; Hwang et al. 2024; Matikainen 2017; Mazzella Ebstein et al. 2020), competing clinical demands (Colwill et al. 2024; Evans et al. 2014; Higgins et al. 2010; Lehmkuhl et al. 2020; Lieschke et al. 2022; Mazzella Ebstein et al. 2020; McLaughlin et al. 2013), high patient acuity (Williams et al. 2024) and patient‐care backlogs (Martin 2017). Nurses could experience challenges juggling research activities with their clinical role (Evans et al. 2014; Hwang et al. 2024; Janerka et al. 2024; Landeen et al. 2017; Snelgrove and James 2011) because nurses are often expected to engage in research on their own time, on top of their clinical role and are not compensated for their time or released from their clinical duties (Jeffs et al. 2013; Matikainen 2017; Walsh et al. 2023). While on shift, they often face heavy workload pressures that prevent them from engaging in research, as reported in 18 articles. When nurses do have release time from their clinical duties to engage in research, there is often a lack of staff coverage to backfill the shifts that they were released from due to staff shortages (Chlan et al. 2020; Janerka et al. 2024; Jeffs et al. 2013; Matikainen 2017; Morrison et al. 2022; Scarsini et al. 2022). Unfortunately, for those nurses who do engage in research, time constraints result in smaller‐scale projects (Chlan et al. 2020) and the inability to keep up to date with research protocols (Brown et al. 2018).

#### 4.2.2 Inadequate Research Experience, Training and Mentorship

The second most reported barrier to nurses' ability to engage in research was a lack of knowledge and understanding of research and the research process, reported in 37 articles. This included limited research skills and experience (Colwill et al. 2024; Davis et al. 2019; Fullam et al. 2018; Higgins et al. 2010; Kelly et al. 2016; Latimer and Kimbell 2010; Lehmkuhl et al. 2020; Mazzella Ebstein et al. 2020; McLaughlin et al. 2013; Morrison et al. 2022; Snelgrove and James 2011; Walden et al. 2015) that was most often due to a lack of formal training (Kelly et al. 2016; Morrison et al. 2022; Nowlin et al. 2021; Ramon et al. 2022; Robichaud‐Ekstrand 2016; Williams et al. 2024), which is a leading cause of nurses' low confidence in conducting research. Specific research skills that nurses often lacked included: preparing and submitting ethics applications, writing funding applications, setting up study procedures, writing research protocols and collecting and analysing data (Alomari et al. 2020; Drury et al. 2024; Engh et al. 2022; Evans et al. 2014; Gallagher et al. 2021; Kelly et al. 2016; Khair et al. 2012; Latimer and Kimbell 2010; Martin 2017; Matikainen 2017; McLaughlin et al. 2013; Nowlin et al. 2021). Oftentimes, this stemmed from a lack of prior research education in the nurses' undergraduate curriculum (Arthur et al. 2019; Chlan et al. 2020; Drury et al. 2024; Janerka et al. 2024; Morrison et al. 2022; O'Brien et al. 2022; Siedlecki and Albert 2017; Snelgrove and James 2011; Weitzel and Robinson 2011). Most nurses' higher level research training occurred during graduate training (Bench et al. 2019; Gonzalez‐de la Torre et al. 2023). Unfortunately, when research training opportunities were offered, attendance could be low (Hwang et al. 2024) and competition was high when the training took the form of fellowships (Twelvetree et al. 2019).

There were a variety of areas in which nurses lacked research training and experience. For example, nurses do not realise or know that research is time‐consuming (McKee et al. 2017), nor do they know how to use research technology and platforms (e.g., quantitative and qualitative data analysis software) (Gullatte and Corwin 2018; Pintz et al. 2018; Robichaud‐Ekstrand 2016). Nurses reported that they were unable to read and understand research articles because they were not familiar with the research terminology and language (Bench et al. 2019; Higgins et al. 2010; Hwang et al. 2024; Martin 2017; Morrison et al. 2022; Robichaud‐Ekstrand 2016). Most nurses do not know how to serve as a Principal Investigator on a study and oftentimes to be a Principal Investigator, nurses must have an adjunct faculty appointment at an academic institution to properly hold and manage the research funding (Gullatte and Corwin 2018). The majority of nurses are not familiar with the research process (Colwill et al. 2024; Gettrust et al. 2016) or have experience navigating institutional processes (Lehmkuhl et al. 2020), including applying for Institutional Review Board approval (Scarsini et al. 2022). Because nurses lack confidence and experience in research, they may be hesitant to discuss research with patients and families (Krewulak et al. 2024). It is particularly difficult for new graduate nurses to engage in research because they are at the beginning phases of transitioning to professional practice and adding research to their role can feel overwhelming (Crozier et al. 2012; Matikainen 2017).

A key barrier to nurses engaging in research is insufficient mentoring capacity (Chlan et al. 2020; Gallagher et al. 2021; Kelly et al. 2016; Kress 2018; Morrison et al. 2022; Robichaud‐Ekstrand 2016; Scala et al. 2016; Snelgrove and James 2011; Walsh et al. 2023; Williams et al. 2024) due to a lack of access to experts (Akerjordet et al. 2012b), research supervision and support (Akerjordet et al. 2012a) and experienced mentors (Drury et al. 2024). Specifically, nurses rarely have access to experienced mentors with advanced research training to receive assistance in formulating a research question (Hwang et al. 2024), proposal writing (Kelly et al. 2016; Pintz et al. 2018; Westlake et al. 2024), drafting ethics applications, study development (Kelly et al. 2016), study implementation (Brown et al. 2018), data analysis (Westlake et al. 2024) and publishing (Khair et al. 2012). Mentorship can be in the form of a dedicated nurse scientist (Pintz et al. 2018), PhD‐prepared nurses working in an organization (Gonzalez‐de la Torre et al. 2023; Jamerson and Vermeersch 2012) or partnerships between the academic and clinical setting (Higgins et al. 2010; McKee et al. 2017; Robichaud‐Ekstrand 2016). However, it is important that mentors not only have advanced research training, but also expertise on the research topic (McKee et al. 2017). When there are academic‐clinical partnerships, tension can arise when there are different expectations and supervision models (Back‐Pettersson et al. 2013).

#### 4.2.3 Negative Internal Beliefs Resulting in a Lack of Motivation to Engage in Research

Nurses can hold negative attitudes towards engaging in research (Bohman et al. 2013; Brown et al. 2018; Farquharson 2024; Matikainen 2017) and be sceptical (Higgins et al. 2010), which results in a lack of motivation or desire to do or engage in clinical research (Khair et al. 2012; Pepper et al. 2024; Walsh et al. 2023). Most often, nurses lack confidence as a result of the belief that they do not have the skills, knowledge or abilities required to engage in research (Bench et al. 2019; Berthelsen and Holge‐Hazelton 2015, 2017; Carter et al. 2020; Duffy et al. 2015; Khair et al. 2012; Matikainen 2017; Morrison et al. 2022; O'Brien et al. 2022; Pepper et al. 2024; Robichaud‐Ekstrand 2016; Siedlecki and Albert 2017; Snelgrove and James 2011; Walsh et al. 2023). Some nurses do not perceive themselves as having the ability or authority to use knowledge generated from research to implement practice changes (Weitzel and Robinson 2011), initiate a research project or seek funding (Westlake et al. 2024). Some nurses are fearful, anxious and intimidated about the idea of conducting research (Alomari et al. 2020; Bench et al. 2019; Engh et al. 2022; Turjanica et al. 2013; Walden et al. 2015) because the research process can be viewed as complex and onerous (Snelgrove and James 2011). There is a perspective that only nurses with graduate training or PhDs are qualified and positioned to do research (Siedlecki and Albert 2017), along with a negative perception of research due to previous academic experiences (Alomari et al. 2020) or prior research experiences that were frustrating (Martin 2017).

Some nurses simply do not have an interest or desire to engage in nursing research (Akerjordet et al. 2012a; Brown et al. 2018; Fowler and Brown 2020). This can be especially true for senior nurses who are near retirement and do not want to learn new skills (Martin 2017). While other nurses lack motivation due to burnout and lack the energy to invest in learning new research skills (Bench et al. 2019). ‘Good nursing’ can be viewed as providing hands‐on clinical care (Higgins et al. 2010), where research is not perceived as part of the nursing role (Bench et al. 2019; Brown et al. 2018; Colwill et al. 2024; Evans et al. 2014; Higgins et al. 2010; Hwang et al. 2024; Kress 2018; Krewulak et al. 2024; McKee et al. 2017; Shepherd et al. 2022) or relevant to current daily practice (Bohman et al. 2013; Higgins et al. 2010; Kelly et al. 2016). Rather, nursing is considered to be strictly a practice profession and does not need to include research (Gonzalez‐de la Torre et al. 2023). Engaging in clinical research can be viewed as something done by physicians and the medical community (Crozier et al. 2012) and when nurses do engage in research, it is considered a luxury (Higgins et al. 2010). Surprisingly, some nurses do not believe that the nursing profession requires a research‐based knowledge (Gonzalez‐de la Torre et al. 2023) and have the perception that research is imposed on them, with no choice (Fowler and Brown 2020; Higgins et al. 2010).

Nurses may hold misconceptions about the ethical and legal aspects of research. For example, they can view research as burdensome on families during times of stress (Brown et al. 2018) and believe that children should not be research participants (Brown et al. 2018). Also, nurses can be fearful that something could go wrong with the research study (Brown et al. 2018) and worry about the ethical implications, such as consent and equal treatment (Brown et al. 2018; McKee et al. 2017).

Limited opportunities for nurses to implement their research study in the clinical setting and to disseminate their research findings into practice decrease nurses' motivation to engage in research (Back‐Pettersson et al. 2013; Chlan et al. 2020). This results in nurses having no sense of ownership of the study and the findings (Fowler and Brown 2020; Higgins et al. 2010). The fast‐paced clinical environment can result in nurses lacking ambition and motivation to also take on research projects (Bohman et al. 2013). Nurses can be hesitant to invest time into research because they fear they will do all the work to conduct a research study but be unsuccessful at publishing their results (McKee et al. 2017) due to poor writing skills (Siedlecki and Albert 2017). Some nurses who engage in research have feelings of isolation from colleagues who do not engage in research (Back‐Pettersson et al. 2013). Lastly, nurses can feel undervalued due to a lack of recognition and communication about scholarly research work across academic and clinical systems (Gullatte and Corwin 2018; Montgomery et al. 2021): they do not feel nursing research is valued by the organization and therefore, not worth conducting.

#### 4.2.4 Insufficient Support From Organizations and Leadership

Organizational and institutional leaders make operational decisions that determine how clinical nurses allocate their time. A frequently reported barrier to nurses engaging in research was a lack of institutional support (Loke et al. 2014; Montgomery et al. 2021; Morrison et al. 2022; Nowlin et al. 2021; O'Brien et al. 2022; Ramon et al. 2022; Scala et al. 2016; Scarsini et al. 2022). This was demonstrated by inadequate support from leadership (Bench et al. 2019; Bohman et al. 2013; Janerka et al. 2024; Jeffs et al. 2013; Kelly et al. 2016; Robichaud‐Ekstrand 2016; Scarsini et al. 2022; Walsh et al. 2023), nurses' beliefs that their organization did not value nursing research (Bohman et al. 2013; Farquharson 2024) and scarce institutional strategies and opportunities for nursing research (Evans et al. 2014; Scala et al. 2019; Shepherd et al. 2022; Twelvetree et al. 2019; Williams et al. 2024). It was common for studies to report that organizations did not prioritise nurses engaging in research (Higgins et al. 2010; Jeffs et al. 2013; Morrison et al. 2022; Shepherd et al. 2022), but rather prioritized nursing time on direct patient care (Khair et al. 2012; Weitzel and Robinson 2011). Nurses not only faced a lack of support at the leadership and organization level, but also at the unit level from their managers (Fullam et al. 2018; Higgins et al. 2010; Khair et al. 2012; Kress 2018; Lieschke et al. 2022; Martin 2017; O'Brien et al. 2022), where managers prioritized nursing time on short‐term clinical care goals over the long‐term benefits of empowering nurses to gain research skills (Higgins et al. 2010) and viewed research as a potential for disruptions to clinical workflows (Gettrust et al. 2016).

Organizational leadership often provided limited financial support for nursing research (Akerjordet et al. 2012b; Arthur et al. 2019; Chlan et al. 2020; Montgomery et al. 2021). Rarely do nurses receive funding to conduct nursing‐led research studies (Bench et al. 2019; Davis et al. 2019; Higgins et al. 2010; Hwang et al. 2024; Kelly et al. 2016; McLaughlin et al. 2013; Morrison et al. 2022; Pintz et al. 2018; Robichaud‐Ekstrand 2016; Scala et al. 2016; Scarsini et al. 2022; Siedlecki and Albert 2017; Walsh et al. 2023), including small grant competitions (Scala et al. 2016), nor are funds allocated to support nursing research infrastructure needed for nurses to successfully engage in research (Arthur et al. 2019; Engh et al. 2022; Evans et al. 2014; Higgins et al. 2010; Latimer and Kimbell 2010; Lieschke et al. 2022; Loke et al. 2014; McKee et al. 2017; Morrison et al. 2022; Robichaud‐Ekstrand 2016; Stutzman et al. 2016). Such infrastructure includes: library supports (e.g., health sciences librarian and access to research databases) (Bohman et al. 2013; Kelly et al. 2016; Kress 2018; Scarsini et al. 2022), biostatistical support (e.g., biostatisticians) (Hwang et al. 2024; Kelly et al. 2016; Pintz et al. 2018), training and education (Hwang et al. 2024), secretarial support (Kelly et al. 2016), research assistant/coordinator (Hwang et al. 2024; Kelly et al. 2016), research technology (Kelly et al. 2016; Scarsini et al. 2022), data entry staff (Kelly et al. 2016) and release time for nurses (discussed above). When infrastructure is in place, the organization's lack of communication about resources and infrastructure to support nurses' engagement in research is a barrier. When this happens, nurses are not made aware of the opportunities that they have to engage in training programmes and research opportunities (Lehmkuhl et al. 2020; Mendoza et al. 2023; Robichaud‐Ekstrand 2016; Scala et al. 2019; Shepherd et al. 2022), nor are they aware of the nursing research studies that are occurring within their organization (Krewulak et al. 2024). Insufficient communication at the unit level about nursing lead research projects can prevent nurses from being aware of ongoing projects and able to support and engage in them (Fowler and Brown 2020; Gullatte and Corwin 2018; Hwang et al. 2024).

Lack of leadership support for nursing research translates into a lack of support for nurses' professional development. This includes leadership not integrating the role of a researcher into everyday nursing clinical roles (Ramon et al. 2022) or clarifying nurses' clinical roles and research roles (Alomari et al. 2020; McKee et al. 2017; Williams et al. 2024). When nurses do conduct research, it is seldom included, recognized or valued as part of their performance evaluations (Westlake et al. 2024). Some organizations have restrictions on nurses' ability to serve as Principal Investigator on nursing‐led studies; therefore, they are not able to hold research funding (Carter et al. 2020; Stutzman et al. 2016). Other ways organizational leadership can fail to support nurses' professional development via research include: lack of incentives for nurses to participate and engage in research (Brown et al. 2018; Drury et al. 2024; Morrison et al. 2022; Ramon et al. 2022), no structured clinical‐academic career pathway for PhD nurses (Farquharson 2024) and no clear opportunities for nurses to engage in research (Walsh et al. 2023).

#### 4.2.5 Unsupportive Research Culture

The absence of an organizational culture that supports and encourages clinical nursing research creates significant barriers to nurses' participation (Evans et al. 2014; Fowler and Brown 2020; Janerka et al. 2024; Kelly et al. 2016; Morrison et al. 2022; Scarsini et al. 2022). Various factors contribute to a poor research culture within an organization. One is unsupportive peers and colleagues towards nurses conducting research (Alomari et al. 2020; Gallagher et al. 2021; Kress 2018; Morrison et al. 2022), where nurses feel undervalued by their peers and leaders for the work they commit to nursing research (Dupin et al. 2014). Other factors include limited opportunities for nurses to collaborate in research (Arthur et al. 2019), prioritisation of nursing care and clinical care routines (Higgins et al. 2010; Robichaud‐Ekstrand 2016) and resistance to change by staff and/or the larger organization (Dupin et al. 2014).

Clinical nurses are rarely in a position where they have decision‐making power to advocate for funding allowances to engage in research (Bench et al. 2019). However, other disciplines with more decision‐making power, such as physicians, often do not value, support or prioritise nursing research (Farquharson 2024; Pintz et al. 2018). Nurses and organizational cultures can hold the perspective that only physicians do research and witness clinical research dominated by physician‐led research (Kelly et al. 2016; Martin 2017; O'Brien et al. 2022; Snelgrove and James 2011). This is in part because oftentimes physicians have the opportunities, resources and privileges to do research (Loke et al. 2014). To increase nursing research within an organization, a culture shift is required (Shepherd and Quinn 2024).

### 4.3 Facilitators

While numerous barriers impede nurses' ability to engage in research, there are multiple facilitators that enable and support clinical nurses in successfully participating in and leading nursing research. These facilitators represent opportunities for healthcare organizations, nursing leaders and individual nurses to create workplace cultures and environments that foster research engagement and build nursing research capacity. Facilitators to nurses' engagement in clinical research included: (a) research infrastructure, (b) education, training and mentorship, (c) visionary leadership, (d) unit‐level facilitators, (e) motivating factors and (f) organizational culture. See Figure 4 for a representation of the main facilitators and their corresponding references.

**FIGURE 4:**
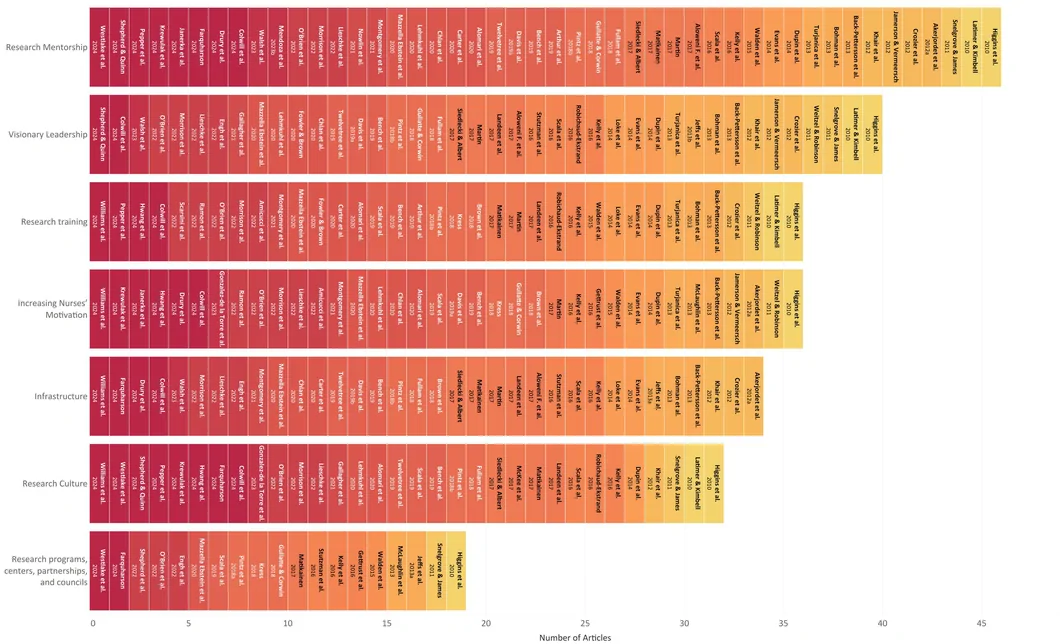
Representation of the main facilitators to nurses' engagement in research and the corresponding references. [Colour figure can be viewed at wileyonlinelibrary.com]

#### 4.3.1 Building Research Infrastructure: Dedicated Nursing Research Programmes and Centres

Visible research programmes and centres within the hospital were effective at creating research‐intensive environments (Farquharson 2024) and increasing nurses' engagement in research (Higgins et al. 2010; Mazzella Ebstein et al. 2020). Examples include: a dedicated director of nursing research (Jeffs et al. 2013), research and development groups (Snelgrove and James 2011), shared governance and research councils (Higgins et al. 2010; Kress 2018; Mazzella Ebstein et al. 2020; Scala et al. 2016; Shepherd et al. 2022; Snelgrove and James 2011; Westlake et al. 2024), a nursing hospital‐based research programme (Kelly et al. 2016), Magnet requirement councils (Pintz et al. 2018), an endowed chair for research funding (Engh et al. 2022), a research office or institute (Scala et al. 2016) and a research unit (Matikainen 2017). It was important to have the presence of these dedicated research staff within the organization (Amicucci et al. 2022; McLaughlin et al. 2013) so that the support services were in close proximity to the clinical nurses (Krewulak et al. 2024). These centres and programmes needed to be flexible and adaptable, able to support the variability in nurses' shift patterns (Jeffs et al. 2013).

Partnerships between the hospital and an academic institution were another way to offer frontline practicing nurses access to research mentorship (Gullatte and Corwin 2018; Higgins et al. 2010; Jeffs et al. 2013; O'Brien et al. 2022; Pintz et al. 2018; Scala et al. 2016, 2019; Stutzman et al. 2016; Walden et al. 2015; Westlake et al. 2024). Within such partnerships, transparency and clear guidelines were required between researchers and institutions (Loke et al. 2014), along with strong leadership to act as a liaison between the academic and clinical sites (Gettrust et al. 2016; Higgins et al. 2010). Organizational policies to support the research process were also required for partnerships to flourish (McLaughlin et al. 2013).

Activities within the organization, oftentimes within the centres and programmes themselves, that helped foster nursing research included: research scholarship programmes (Lieschke et al. 2022; Walden et al. 2015), funded nursing fellowships (Higgins et al. 2010; Scala et al. 2016, 2019; Stutzman et al. 2016), residency programmes (Kelly et al. 2016), a research committee (Carter et al. 2020; Pintz et al. 2018) and monthly fellowship meetings (Carter et al. 2020). Involvement and memberships in societies also helped advance nursing research (Gonzalez‐de la Torre et al. 2023), such as the Theta Tau chapter (Weitzel and Robinson 2011). It was important for nurses to be in a position to be a Principal Investigator (PI) on their own research projects (Scala et al. 2016), which could be accomplished through university adjunct positions or academic‐clinical partnerships. The organization needs to have supportive research infrastructure (Mazzella Ebstein et al. 2020; Scala et al. 2016), including: funding and support for preparation of grant proposals (Akerjordet et al. 2012a; Crozier et al. 2012; Stutzman et al. 2016), library services and resources (access to databases and librarian) (Bohman et al. 2013; Landeen et al. 2017; Pintz et al. 2018; Scala et al. 2016; Siedlecki and Albert 2017), budget support (Kelly et al. 2016; Loke et al. 2014), administrative assistance (Kelly et al. 2016), computer software (Kelly et al. 2016), research material expenses (Kelly et al. 2016), support with data entry, analysis and management (Kelly et al. 2016; Scala et al. 2016), biostatistical support (Carter et al. 2020; Landeen et al. 2017; Lieschke et al. 2022; Scala et al. 2016; Siedlecki and Albert 2017) and manuscript writing (Carter et al. 2020). These services could be offered in the form of consultations (Stutzman et al. 2016).

The most reported infrastructure requirement for nurses to engage in research was protected and dedicated paid research time (Back‐Pettersson et al. 2013; Bench et al. 2019; Brown et al. 2018; Carter et al. 2020; Chlan et al. 2020; Colwill et al. 2024; Crozier et al. 2012; Drury et al. 2024; Engh et al. 2022; Evans et al. 2014; Farquharson 2024; Fullam et al. 2018; Kelly et al. 2016; Khair et al. 2012; Loke et al. 2014; Martin 2017; Mazzella Ebstein et al. 2020; Montgomery et al. 2021; Morrison et al. 2022; Pintz et al. 2018; Scala et al. 2016; Twelvetree et al. 2019; Walsh et al. 2023). This could take the form of adapted work hours (Akerjordet et al. 2012a) and manager‐approved release time (Matikainen 2017), with scheduling support (Williams et al. 2024). For example, release time from a clinical shift 1 day/week to work on research (Aloweni et al. 2017). Since clinical research is costly, nurses need awareness and access to sufficient funding opportunities from internal and philanthropic streams to conduct the research (Chlan et al. 2020; Crozier et al. 2012; Davis et al. 2019; Jeffs et al. 2013).

#### 4.3.2 Cultivating Nurse Research Champions: The Power of Education, Training and Mentorship

When nurses are given opportunities to learn about research, attend educational sessions and gain experience in non‐intimidating and supportive environments (Pintz et al. 2018; Scala et al. 2016; Turjanica et al. 2013), they are more likely to conduct research at the bedside. Research training can be accomplished via structured research education (Alomari et al. 2020; Arthur et al. 2019; Bench et al. 2019; Brown et al. 2018; Colwill et al. 2024; Crozier et al. 2012; Evans et al. 2014; Higgins et al. 2010; Hwang et al. 2024; Kelly et al. 2016; Latimer and Kimbell 2010; Martin 2017; Montgomery et al. 2021; Morrison et al. 2022; O'Brien et al. 2022; Ramon et al. 2022; Scala et al. 2019; Williams et al. 2024). These opportunities need to be provided by experts (Landeen et al. 2017), flexible and offered at central locations with an option for online attendance (Bench et al. 2019; Engh et al. 2022; Hwang et al. 2024). Examples of learning opportunities includ: nursing research fair (Turjanica et al. 2013), education sessions (Higgins et al. 2010), one‐on‐one coaching (Kelly et al. 2016), workshops (Arthur et al. 2019; Bench et al. 2019; Carter et al. 2020; Crozier et al. 2012; Pepper et al. 2024), peer‐to‐peer discussion (Kress 2018) and a research training course (Amicucci et al. 2022; Kress 2018), often in the form of an online module (Kress 2018).

Education should be tailored to clinical nurses' needs (Mazzella Ebstein et al. 2020), practical (Snelgrove and James 2011) and adjusted to meet varying knowledge levels (Weitzel and Robinson 2011). Topics should include: learning about the research process (i.e., research questions, protocol/proposal development, IRB, implementation, monitoring, analysis and evaluation) (Crozier et al. 2012; Fowler and Brown 2020; Kelly et al. 2016; Scala et al. 2016), research methods (Martin 2017), literature searching skills (Akerjordet et al. 2012a), research literacy skills (Evans et al. 2014) and writing and publishing (Carter et al. 2020; Khair et al. 2012; Pepper et al. 2024; Scala et al. 2016). This can be accomplished by implementing journal clubs (Bench et al. 2019; Colwill et al. 2024; Higgins et al. 2010; Kress 2018; Matikainen 2017) where nurses regularly read, engage with and discuss research findings from scientific journals (Amicucci et al. 2022; Scarsini et al. 2022). Nurses appreciated when the learning was fun and hands‐on (Dupin et al. 2014; Loke et al. 2014; Walden et al. 2015), includes open discussion (Robichaud‐Ekstrand 2016), fosters reflection, encourages peer learning (Fowler and Brown 2020), is interactive and participatory in nature (Weitzel and Robinson 2011) and involves peer‐to‐peer support (Back‐Pettersson et al. 2013). Nurses also want opportunities for continuing education (Bohman et al. 2013; Kress 2018; Montgomery et al. 2021) and for nurses who are taking advanced degrees, reimbursement for tuition (Pintz et al. 2018).

Having access to mentorship and support from experienced/senior researchers was a frequently reported facilitator that supported nurses' training and built their confidence, reported in 46 articles. Mentorship was a way nurses could learn about the research process from an experienced nurse researcher who clinical nurses viewed as a role model (Akerjordet et al. 2012a; Jamerson and Vermeersch 2012; Siedlecki and Albert 2017). It was important that there was a clear pathway to receive mentorship from PhD prepared nurse scientists (Arthur et al. 2019; Kelly et al. 2016; Morrison et al. 2022; Scala et al. 2016). Ways this mentorship could be delivered included: structured mentorship meetings (Chlan et al. 2020; Colwill et al. 2024), small group coaching (Gullatte and Corwin 2018) and mentorship programmes (Pintz et al. 2018). The mentors needed to be visible and purposefully make themselves available to assist when needed (Bench et al. 2019; Scala et al. 2016; Shepherd et al. 2022). It was helpful if the mentor was a full‐time employee within the clinical institution (Higgins et al. 2010; Jamerson and Vermeersch 2012), a designated nurse scientist (Pintz et al. 2018), someone with track record of successful funding (Farquharson 2024) and able to identify research areas (Akerjordet et al. 2012a). Mentorship was key to learning because mentors walked alongside clinical nurses as they actively conducted research and assisted them in gaining hands‐on experience (Bohman et al. 2013; Drury et al. 2024; Evans et al. 2014), including supporting them through the IRB process (Scala et al. 2016). Mentors could also allow nurses to shadow them (Bench et al. 2019) or participate in larger nursing‐led studies (Khair et al. 2012).

#### 4.3.3 Visionary Leadership: Empowering Nurses' Research Engagement

Organizational support and endorsement from nurse managers and senior leadership were facilitators of nurses' engagement in research, reported in 40 articles. This included strategic alignment with hospital priorities (Engh et al. 2022; Jeffs et al. 2013; Kelly et al. 2016; Scala et al. 2016; Snelgrove and James 2011), strong stakeholder engagement (Jeffs et al. 2013), Chief Nursing Officer (CNO)/Chief Executive Officer (CEO) support and commitment to research (Jamerson and Vermeersch 2012; Kelly et al. 2016; Scala et al. 2016) and strategic buy‐in from senior managers and academic leaders (Twelvetree et al. 2019). It was important for leadership support, from both hospitals and academic institutions, to be visible and transparent for clinical nurses to see (Bench et al. 2019; Crozier et al. 2012; Davis et al. 2019; Dupin et al. 2014; Engh et al. 2022; Fowler and Brown 2020; Gullatte and Corwin 2018; Higgins et al. 2010; Landeen et al. 2017; Latimer and Kimbell 2010). One way to make leadership support visible was for managers at the unit level to support and encourage nurses to lead and engage in research (Bohman et al. 2013; Gallagher et al. 2021; Higgins et al. 2010; Khair et al. 2012; Loke et al. 2014; Martin 2017; Siedlecki and Albert 2017). This type of leadership environment was often present among hospitals with an emphasis on meeting Magnet designation (Chlan et al. 2020; Kelly et al. 2016; McLaughlin et al. 2013). One way hospital organizations could demonstrate strong leadership in support of nursing research was to provide clear communication about research opportunities (Colwill et al. 2024; Evans et al. 2014; Fowler and Brown 2020) and have physicians on board to support nursing research (Loke et al. 2014).

#### 4.3.4 Unit Level Facilitators

There were unit‐level factors that facilitated nurses' engagement in research. These included implementing nurses who acted as a research champion (Engh et al. 2022; Krewulak et al. 2024; Scala et al. 2016; Shepherd et al. 2022), connecting research projects with ongoing EBP activities (Scala et al. 2016) and integrating research tasks into existing daily workflows (Fowler and Brown 2020; Gettrust et al. 2016; Ramon et al. 2022). It was important to have nurses conducting research that was relevant and applicable to the unit‐level context (Alomari et al. 2020; Aloweni et al. 2017; Brown et al. 2018; Dupin et al. 2014; Evans et al. 2014; Gettrust et al. 2016; Gullatte and Corwin 2018; Hwang et al. 2024; Lieschke et al. 2022; Scala et al. 2016, 2019; Snelgrove and James 2011; Williams et al. 2024), asking research questions and conducting research projects that addressed nurses' clinical concerns (Dupin et al. 2014; Lieschke et al. 2022), problems (Janerka et al. 2024) and topics of interest (Scala et al. 2019), while also being aligned with nurses' clinical roles (Aloweni et al. 2017). Also, it was important that nurses conducting the research were familiar with and understood the unit where the research was conducted, because this helped build the staff nurses' trust in the research team (O'Brien et al. 2022).

Nurses were motivated to engage in research when there were positive outcomes from the research (Fowler and Brown 2020; O'Brien et al. 2022), changes were beneficial for patient care (Gettrust et al. 2016; Morrison et al. 2022) and they received positive feedback from families, validating the care they received (Fowler and Brown 2020). Specifically, research that produced findings that were translatable to practice (Back‐Pettersson et al. 2013; Krewulak et al. 2024; Matikainen 2017; Morrison et al. 2022) and had a clear focus on improving care helped ignite nurses' passion for continuing to engage in research (Lieschke et al. 2022).

#### 4.3.5 Making Research Matter: Motivating Factors

Nurse were motivated to engage in research and found fulfilment doing so when they were given the opportunities to engage in all research processes, including funding support for dissemination (Chlan et al. 2020; Davis et al. 2019; Gonzalez‐de la Torre et al. 2023; Gullatte and Corwin 2018; McLaughlin et al. 2013; Montgomery et al. 2021; Morrison et al. 2022). Dissemination activities included: support to present research at conferences (i.e., internal, national and international conferences) (Back‐Pettersson et al. 2013; Bench et al. 2019; Colwill et al. 2024; Gonzalez‐de la Torre et al. 2023; Martin 2017; Turjanica et al. 2013), invitations to present study findings at Nursing Grand rounds within hospital organizations (Walden et al. 2015), research project displayed in newsletters and the hospital library displays (Weitzel and Robinson 2011), journal publication (Gonzalez‐de la Torre et al. 2023; Krewulak et al. 2024) and communication of findings to those involved (researchers and participants) (Brown et al. 2018; Gonzalez‐de la Torre et al. 2023).

There were various internal motivators that propelled nurses to engage in research, including those who had a desire to develop new skills (Janerka et al. 2024), personal locus of control (Colwill et al. 2024), positive attitudes towards research (Bohman et al. 2013), passion to improve patient care (Morrison et al. 2022; Williams et al. 2024), professional curiosity (Colwill et al. 2024; Lehmkuhl et al. 2020), recognition that research improves patient outcomes (Brown et al. 2018), personal experience of a family member in the hospital (Colwill et al. 2024) and the ability to see a purpose and value in research (Akerjordet et al. 2012b). Other motivating factors included organizational incentive (Drury et al. 2024; Lieschke et al. 2022; Ramon et al. 2022; Scala et al. 2019) and a clinical trigger event that prompted inquiry (Morrison et al. 2022; Siedlecki and Albert 2017). Nurses were propelled to engage in research when they experienced incremental success that built their confidence and gave them a sense of ownership, because this made them feel valued and appreciated for their contributions to research (Alomari et al. 2020; Gettrust et al. 2016; Higgins et al. 2010; Mazzella Ebstein et al. 2020; O'Brien et al. 2022). This could be accomplished by recognizing nurses for their work and research involvement (Brown et al. 2018; Evans et al. 2014; Krewulak et al. 2024; Lieschke et al. 2022; Ramon et al. 2022; Scala et al. 2016; Siedlecki and Albert 2017), especially when improvements were made to clinical care (Krewulak et al. 2024), manuscripts were accepted for publication (O'Brien et al. 2022) or a nurse was highlighted in the media (Turjanica et al. 2013). Nurses could experience personal benefits from engaging in research (Evans et al. 2014), such as increased job satisfaction (Janerka et al. 2024; Scala et al. 2019) and feeling empowered to make a difference in their clinical setting (Morrison et al. 2022; Scala et al. 2019).

One commonly noted factor that motivated nurses to engage in research was whether it could help advance their professional development (Colwill et al. 2024; Dupin et al. 2014; Kelly et al. 2016; Kress 2018; O'Brien et al. 2022; Scala et al. 2019). Ways that nursing research supported nurses' professional development included: continuing education credits (Engh et al. 2022), career and professional advancement (Janerka et al. 2024; Morrison et al. 2022; Scala et al. 2019), job promotion (O'Brien et al. 2022) and enhancing the nurses' role to include research activities (Kelly et al. 2016). Nurses appreciated the professional networking opportunities that accompanied engaging in research because this enabled them to collaborate with other nurses who had similar clinical interests from other organizations (Chlan et al. 2020; Gallagher et al. 2021; Khair et al. 2012; Mendoza et al. 2023; O'Brien et al. 2022; Scala et al. 2019). For some nurses, engaging in research encouraged them to pursue higher education (Pintz et al. 2018), while for others, they believed it increased the credibility of the nursing profession (Scala et al. 2019).

Various individual characteristics of nurses were found to influence their engagement in research. Nurses who held an advanced degree (Amicucci et al. 2022; Arthur et al. 2019; Bohman et al. 2013; Drury et al. 2024; Evans et al. 2014; Hwang et al. 2024; Jamerson and Vermeersch 2012; Kelly et al. 2016), including a master's, graduate studies or doctorate and were in non‐direct care roles (e.g., administration, manager or clinical specialist) (Amicucci et al. 2022; Arthur et al. 2019; Colwill et al. 2024; Drury et al. 2024) were more likely to engage in research. Also, nurses who were male (Amicucci et al. 2022), had a personal commitment to improving patient care (Siedlecki and Albert 2017) and were curious, self‐confident and eager to learn (Morrison et al. 2022) were more likely to engage in research.

#### 4.3.6 Building Organizational Cultures That Inspire and Support Nursing Research

During data analysis it was evidence that it was imperative for nurses to work in a research supportive culture, where nursing research was valued, highlighted and encouraged, at both the administrative and unit level (Bench et al. 2019; Gallagher et al. 2021; Higgins et al. 2010; Kelly et al. 2016; Lieschke et al. 2022; Pintz et al. 2018; Siedlecki and Albert 2017; Snelgrove and James 2011; Westlake et al. 2024). There were multiple factors that fostered a research supportive culture, including collaborative multi‐disciplinary research teams (Alomari et al. 2020; Farquharson 2024; Fullam et al. 2018; Gonzalez‐de la Torre et al. 2023; Higgins et al. 2010; Khair et al. 2012; Latimer and Kimbell 2010; McKee et al. 2017; Morrison et al. 2022; Pintz et al. 2018; Scala et al. 2019; Snelgrove and James 2011; Twelvetree et al. 2019). This helped research teams share expertise (Higgins et al. 2010) and collaborate across departments and professional roles (Landeen et al. 2017; Latimer and Kimbell 2010). Another way to gain multi‐disciplinary involvement was to incorporate research project discussions within multi‐disciplinary rounds (Krewulak et al. 2024). Within the research team itself, having positive dynamics and good communication among team members were important (Hwang et al. 2024). Nurses appreciated having support from their peers when conducting research (Bench et al. 2019; Colwill et al. 2024; Matikainen 2017; Shepherd and Quinn 2024; Williams et al. 2024), which could be in the form of peer involvement (Siedlecki and Albert 2017) and a network of peers who engage in research (Pepper et al. 2024).

Supportive research cultures were described as having the following traits: active learning, a spirit of excellence, inquiry (Kelly et al. 2016; Latimer and Kimbell 2010; Matikainen 2017), critical thinking (Dupin et al. 2014), problem solving (Morrison et al. 2022) and respect (Alomari et al. 2020; Morrison et al. 2022). Organizations that openly recognized the value of research for enhancing patient care (Lehmkuhl et al. 2020; Westlake et al. 2024), regularly communicated about nursing research activities (O'Brien et al. 2022), encouraged nurses to pursue advanced degrees (Scala et al. 2016) and normalized nursing research in everyday practice (Landeen et al. 2017; Robichaud‐Ekstrand 2016) fostered positive research cultures.

## 5 Multi‐Level Framework of Barriers and Facilitators

Based on the synthesized findings, we developed a multi‐level framework that depicts how barriers and facilitators at four levels (i.e., individual, unit, organizational and system) influence nurses' engagement in clinical research (see Figure 5). At the individual level, nurses' research skills and level of education interact with their personal motivations and professional commitments to improving care. At the unit level, managers' support, research champions, staffing patterns and the priority placed on research in daily practice can either facilitate or hinder nurses' ability to conduct clinical research. At the organizational level, a formal research infrastructure and strategic priorities that foster a culture of research determine if nursing research excellence is a focus within the organization. Finally, system‐level factors, such as academic partnerships with mentorship and nurses' access to funding and regulatory approval, greatly affect the success of clinical nurses in formally developing and implementing a research project. Arrows in the framework illustrate that the four levels are dynamic and interdependent rather than linear. They influence one another in their ability to either support or hinder nurses' engagement in research. For example, the provision of paid release time at the organizational level can be hindered if not implemented at the unit level, with nurse managers' support and appropriate staffing levels.

**FIGURE 5:**
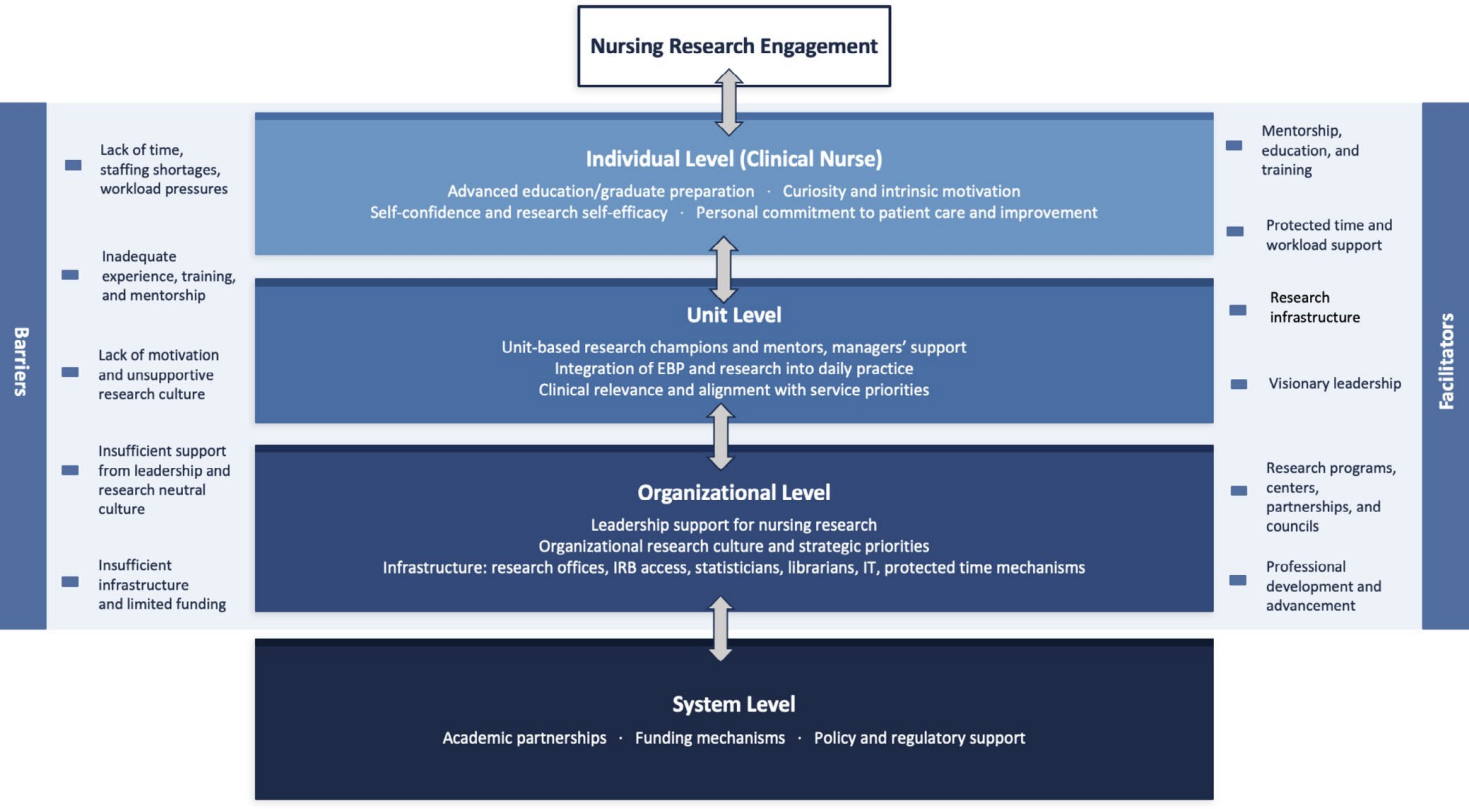
Multi‐level framework of barriers and facilitators influencing nurses' engagement in clinical research. [Colour figure can be viewed at wileyonlinelibrary.com]

## 6 Strategic Recommendations for Building Nursing Research Excellence

Some authors of included articles made future recommendations based on their findings. These recommendations included strong visionary leadership (Akerjordet et al. 2012a; Back‐Pettersson et al. 2013; Bench et al. 2019; Engh et al. 2022; Jeffs et al. 2013; Kelly et al. 2016; Scala et al. 2019) and organizational support (Back‐Pettersson et al. 2013; Jeffs et al. 2013) that values and prioritizes nursing research (Higgins et al. 2010; Lehmkuhl et al. 2020; Matikainen 2017) and creates a positive research culture within the institution (Akerjordet et al. 2012a; Bench et al. 2019; Drury et al. 2024; Janerka et al. 2024; Kelly et al. 2016; Walden et al. 2015; Walsh et al. 2023). Leadership that recognizes and acknowledges nurses' scholarly contributions (Engh et al. 2022), with managers who create environments where nurses feel inspired to engage in research (Akerjordet et al. 2012b, 2012a; Amicucci et al. 2022; Brown et al. 2018; Morrison et al. 2022). To do this, leadership should conduct a comprehensive assessment of the research infrastructure (Montgomery et al. 2021) and develop various programmes and opportunities, such as research training and education (Akerjordet et al. 2012b; Bohman et al. 2013; Brown et al. 2018; Drury et al. 2024; Gettrust et al. 2016; Kelly et al. 2016; Kress 2018; Krewulak et al. 2024; Mazzella Ebstein et al. 2020; Ramon et al. 2022; Scala et al. 2019; Williams et al. 2024), mentorship and research supervision (e.g., fellowship programmes, scholarships, internship programmes) (Arthur et al. 2019; Drury et al. 2024; Gallagher et al. 2021; Kelly et al. 2016; Lieschke et al. 2022; Matikainen 2017; Mazzella Ebstein et al. 2020; Robichaud‐Ekstrand 2016; Walsh et al. 2023; Williams et al. 2024) and protected and paid release time for nurses to do research (Akerjordet et al. 2012b; Gonzalez‐de la Torre et al. 2023; Kelly et al. 2016; Landeen et al. 2017; Mazzella Ebstein et al. 2020; Walsh et al. 2023; Williams et al. 2024).

The development of clinical‐academic partnerships should be explored, because they are a beneficial way to increase mentorship and training among clinical nurses (Arthur et al. 2019; Back‐Pettersson et al. 2013; Landeen et al. 2017; Robichaud‐Ekstrand 2016). When research does take place, leadership should communicate this to nurses to increase the visibility of research projects within organizations (Brown et al. 2018) and ensure patients are involved in nursing research to increase the relevance of the project (Robichaud‐Ekstrand 2016). Funding should be established for both small and large scale studies (Higgins et al. 2010; Jeffs et al. 2013) and increased infrastructure developed that includes education, multi‐disciplinary collaboration, networking opportunities, financial support, translation of research findings and research assistant support for nurses (Alomari et al. 2020; Amicucci et al. 2022; Arthur et al. 2019; Back‐Pettersson et al. 2013; Higgins et al. 2010; Krewulak et al. 2024; Lieschke et al. 2022; Robichaud‐Ekstrand 2016). Overall, nurses of today need to work within organizations that are dedicated to the provision of high‐quality and safe care to patients and families by providing nurses with opportunities to gain experience conducting and engaging in clinical research (Alomari et al. 2020).

## 7 Interventions Geared to Increase Nurses' Engagement in Clinical Research

Twenty‐three of the 79 included articles involved an innovation or intervention implemented among clinical nurses aimed at increasing their engagement with research. In one article, nurses participated in an action research team for a nurse‐led study (Alomari et al. 2020). Eight articless involved the development of a programme that took a multi‐modal approach, with a combination of activities geared at increasing nurses' engagement in research (e.g., release time from clinical shifts, education and mentorship) (Aloweni et al. 2017; Back‐Pettersson et al. 2013; Chlan et al. 2020; Crozier et al. 2012; Fullam et al. 2018; McKee et al. 2017). Two of the mentioned programmes included a fellowship (Shepherd et al. 2022; Stutzman et al. 2016), while two interventions were solely a fellowships where nurses were educated and supported to learn about the research process and conduct a research project (Carter et al. 2020; Mazzella Ebstein et al. 2020). Two articles included the development of an academic‐practice partnership between a hospital organization and an academic school of nursing, which was accompanied by faculty mentorship (Davis et al. 2019; Shepherd and Quinn 2024). One academic‐practice partnership involved the hiring of a paid clinician–scientist (Gullatte and Corwin 2018). Other innovations included the implementation of various paid positions, including: research chair (Engh et al. 2022), nurse research facilitator (Jamerson and Vermeersch 2012) and director of nursing (Jeffs et al. 2013). Another innovation involved research training for nurses who were pursuing a master's degree (Landeen et al. 2017), another utilized a nursing research council (Turjanica et al. 2013) and another supported post‐doctoral clinical academics within the hospital organization to conduct research (Twelvetree et al. 2019). Finally, some authors included fun activities to increase nurses' participation in learning about and engaging in research, including: a research workshop (Nixon et al. 2013), a research initiative using cookies to teach the research process (Walden et al. 2015) and holding a nurses' research week (Weitzel and Robinson 2011). See Table 2 for a presentation and description of the various innovations and interventions identified from the included articles.

**TABLE 2:** Interventions to increase clinical nurses' engagement in research.

Author	Intervention	Description
Alomari et al. (2020)	Active participation in an action research project on building the capacity of clinical nurses	Nurses actively participated in the research process of an action research project by being part of an Action Research Team (ART).
Aloweni et al. (2017)	Nursing research immersion programme	Nurses applied to participate in the programme. Nurses were given protected time of 1 day/week for 24 weeks to work on a research topic. One‐on‐one mentorship was provided by PhD/DNP‐prepared nurses throughout the programme. Nurses learned the research process, writing proposals and the ethics approval process. Nurses collected data, conducted analysis and wrote a report.
Back‐Pettersson et al. (2013)	Research & Development Programme	A 2‐year programme for baccalaureate‐prepared registered nurses willing to pursue an academic education for a master's degree. Nurses conducted and presented a research project from idea to publication and participated in national and international research seminars and workshops. Education and coaching were provided as needed, including by a librarian, an in‐house coach and engagement of internal and external senior researchers in the programme.
Carter et al. (2020)	Academic‐practice Research Fellowship	The fellowship emerged from discussions among faculty jointly appointed between a school and a practice setting and nursing leaders (Dean and Associate Dean at the school and the Chief Nursing Officer and Director of Nursing Research at the hospital). Clinical nurses apply once per academic year. The aim of the fellowship was to facilitate clinical nurses in conducting research. Nurses received: (a) one‐on‐one mentorship from nursing faculty, (b) statistical support, (c) didactic instruction in the dissemination of findings, (d) hands‐on guidance in the dissemination of findings, (e) a peer‐reviewed manuscript writing workshop and (f) oversight from the Director of Academic‐Practice Partnerships. Nurses receive protected time to conduct their research: 2 shifts/month in year 1 and 1 shift/month in year 2. They met biweekly with their faculty mentor.
Chlan et al. (2020)	Clinical Nurse Scholar Programme	The programme was developed and implemented by the Associate Dean for Nursing Research at a large Magnet designated health institution. The goal was to provide professional career development opportunities for clinical registered nurses. Nurses receive a 0.2 FTE (1 day/week) clinical release time to actively participate in mentored research for up to 2 years. Nurses apply to the programme and meet bimonthly with their mentor. Opportunities to meet with a medical librarian are provided. Nurses complete online training modules, conduct a literature review, identify knowledge gaps, develop a research protocol, submit for IRB approval and conduct a clinical study by collecting data and analysing findings, along with dissemination. They also receive training in public speaking and writing skills.
Crozier et al. (2012)	The Innovations in Nursing and Midwifery Practice Project (INMPP)	The INMPP consisted of a steering group responsible for developing and instituting infrastructure and processes to support nurse‐led innovation projects, with the intent of developing research capabilities and capacity. The INMPP funded and supported nurse‐ or midwifery‐led innovation projects with a clear patient‐centred focus. Nurses attended individual and group workshops and members of the steering group acted as mentors and provided support.
Davis et al. (2019)	Academic‐Practice Partnership	The partnership began with senior leaders from an academic institution and a healthcare system, with the goal of improving health through nursing practice. The goal was to increase research collaboration and mentorship, consultation and mentoring activities. The partnership provided research collaboration and mentoring through engagement between faculty and staff nurses. Nurses and faculty were paired where they identified a research problem. The faculty provided didactic research training, IRB support and writing assistance. Faculty received 25% workload effort release to oversee the projects. Research projects received funding between $7 and 20K.
Engh et al. (2022)	Hospital‐Based Endowed Chair in Nursing Research	Conversations were held between a nursing research chair and nurses and allied healthcare professionals, who could attend a session to discuss potential research ideas. Topics include an ongoing study, a proposed study, a theoretical issue or an idea for a study. The chair engaged nurses and allied healthcare professionals in discussions about research, providing opportunities to present ongoing research within the hospital.
Fullam et al. (2018)	Research Excellence Across Clinical Healthcare (REACH)	Developed between a regional Nursing & Midwifery Planning & Development unit, nursing groups and an academic institute. Objectives include: (a) identification and initiation of potential clinical research, (b) direct support of small research projects, (c) formation of a network between clinical and academic staff and (d) research skill development. Activities included: (a) one‐on‐one training and support in research design and scientific writing, (b) three skills development workshops, (c) two conferences and (d) formation of steering groups to support two projects.
Gullatte and Corwin (2018)	One Emory Nursing Initiative	This initiative was initiated by the dean of a school of nursing and the chief nursing executive at a healthcare organization. Full‐time paid position for a director of nursing innovation and research was implemented, with an adjunct faculty appointment. This position mentors nurses in the research process and supports them through research question development, methodology, proposal writing and IRB submission, as well as coaching on manuscript publication. Implementation of a 2‐day hands‐on Emory Nursing Evidence‐Based Practice and Research Academy, where nurses learn about quantitative and qualitative research, engage in small group coaching and have a polished research question ready for review of the literature, protocol development and IRB submission. Faculty from the SON act as mentors for these projects.
Jamerson and Vermeersch (2012)	Nurse Research Facilitator	Three responsibilities: conduct research, develop research capacity and build a culture of research. Conducting research can be the facilitator's own research, but most often involves assisting clinical staff with their research studies. Research capacity includes educating staff, consulting with the agency, leading meetings, maintaining records and acting as a liaison. Research culture encompasses strengthening an organization's values and beliefs regarding research. This can involve the development of research agendas/programmes, the designation of specific and other resources and the implementation of efforts to motivate staff.
Jeffs et al. (2013)	Research capacity building blueprint	A director of nursing research was hired at a large Canadian hospital to lead the development, execution and evaluation of a research capacity‐building action plan. Strategies included: (a) providing research and advancing practice formal learning programmes, (b) building infrastructure and research positions, (c) mentoring research interns and trainees, (d) hosting research knowledge translation forums, (e) communicating nursing research activities, (f) establishing a research governance structure and (g) offering consultation services and workshops.
Landeen et al. (2017)	Hope Research Community of Practice (HRCoP)	Seven master's‐prepared nurses at a hospital were invited to participate. They engaged in monthly 3‐h seminars facilitated by 2 PhD‐prepared nurses that covered the research process, writing a research question, choosing a method, obtaining ethics approval, data collection and analysis and planning for dissemination. Nurses received funding for study activities including reimbursements, transcription services and data collection and analysis tools.
Mazzella Ebstein et al. (2020)	Nursing Research Fellowship	Nurses receive 18 days of protected class time and research days over 9 months. Nurses attend classes and research days where they work on writing their study protocol and application to the IRB. Nurses complete online modules and in‐person didactic sessions, along with receiving mentorship from a nurse‐scientist to complete the project. Nurses present their work internally and externally, along with draft manuscripts for publication.
McKee et al. (2017)	Intervention to increase clinical nurse research participation	The intervention aimed to increase research capacity in advanced clinical nursing in an acute urban hospital setting. It included skill development, support for quantitative methodology and assistance from experienced researchers in conducting research studies and disseminating results. Key components included leadership, a steering group, funding, small research groups with support from post‐doctoral fellows, clinical and academic nurse researchers, focused clinical research questions, peer mentorship, experiential learning and education sessions. Mentorship included statistical analysis, study design, academic writing and submission of papers for publication and practice development.
Nixon et al. (2013)	Research Workshop	A needs assessment was conducted, which resulted in the development of a series of workshops for each stage of the research process, to which all nurses and members were invited. At the workshops, consensus was reached to advance two research questions as a trust‐wide research project. Subsequent workshops focused on drafting questionnaires, attaining ethical approval, data collection, data entry and analysis and dissemination of results.
Shepherd et al. (2022)	Strategies to increase staff awareness and engagement in clinical research	Strategies include: (a) Chief Nurse Research Fellows, where clinical staff undertake research training and identify local clinical research priorities (nurses received 1 day/week release for 6 months to work on a project), (b) development of the Embedding Research in Care Unit (an iterative consultation process), (c) Research Facilitator (new role to support research through training and identifying research priorities 1 day per week for 12 months) and (d) development of the Clinical Academic Network so nurses and allied healthcare professionals can collaborate on research.
Shepherd and Quinn (2024)	Research Strategy Partnership	Development of a research strategy framework by a organizations Chief Nursing Operator, assistant director of nursing and a professor of nursing from a university. Three cohorts, a total of 17 nurses, completed the CNRF programme. See above Shepherd et al. (2022) for description.
Stutzman et al. (2016)	Neuroscience Nursing Research Center (NNRC) and Fellowship	The programme is designed for bedside registered nurses to lead their own research study from inception to publication. Nurses apply to the fellowship and receive support to initiate a protocol and follow a timeline. They engage in online research training modules and write a protocol. They receive frequent mentorship to conduct a literature review, develop a research question and attend an 8‐h research boot camp, where they finalise their protocol and submit it for IRB approval. They then collect data, receive mentorship from a scientific writer in statistical analysis and interpretation and draft and submit manuscripts. The NNRC also offers all nurses 1 h consultation meetings as needed to assist with research.
Turjanica et al. (2013)	Dedicated Neuroscience Nursing Research Fair	Three hospitals developed a nursing research council, which initiated a nursing research poster fair for the hospitals. At the fair, nurses showcase nurse‐driven research from the hospitals and local nursing community, along with sharing educational materials to increase nurses knowledge about research
Twelvetree et al. (2019)	Practice‐based approach to support post‐doctoral clinical academics	Aimed to bridge the gap between academia and clinical practice by developing a practice‐based approach to supporting post‐doctoral clinical academics to remain in their clinical roles while allowing them to expand their research skills and those of others. Senior managers supported the incorporation of an action‐learning framework to develop and fund a research project addressing a clinical priority.
Walden et al. (2015)	Great American Cook Experiment (GACE)	The aim was to engage staff nurses in research in an informative and fun way by determining their preference between sugar‐free chocolate chip cookies and regular chocolate chip cookies. A research scholars programme was created to teach the research process to nurses and encourage them to design and conduct research projects under the mentorship of a PhD‐prepared research faculty. GACE was used throughout the curriculum to teach basic research concepts.
Weitzel and Robinson (2011)	Nursing Research Week	Staff nurses on the Nursing Research Council developed and implemented Nursing Research Week to increase nurses' awareness, engagement with and understanding of nursing research. Day 1 was the annual research day conference; day 2 was static poster viewing, day 3 was meet the nursing research council; day 4 was the great cookie caper and day 5 was the research results.

Importantly, across the 23 interventions, most adopted the approach depicted in the multi‐level framework (Figure 5). They targeted facilitators spanning the individual, unit organizational and system levels. For example, combining system‐level support for funding and academic partnerships, organizational‐level support for research infrastructure, unit‐level support from managers and individual‐level support for professional development. These multi‐level approaches resulted in comprehensive interventions to support nurses' engagement in research. Future research should evaluate the impact and outcomes of these interventions to determine the potential best strategies.

## 8 Discussion

In this article, evidence on factors that influence nurses' engagement in clinical research was mapped and synthesized. A total of 79 articles were included and descriptions of barriers and facilitators were provided. The most prominent barriers to clinical nurses engaging in research were a lack of protected time and inadequate training and mentorship. This resulted in nurses often feeling unprepared and unable to conduct clinical research. Other barriers included a lack of motivation, insufficient support from leadership and an unsupportive organizational research culture. Although numerous barriers impeded nurses' engagement in research, there were prominent facilitators that supported and created research‐intensive environments within healthcare organizations. These included strong visionary leadership that built nursing research infrastructure, intentional development of a culture of nursing research and inquiry and investment in programmes, centres and fellowships that afforded nurses the opportunity to attain research training, experience and mentorship. The facilitators and barriers are not isolated factors. Rather, they interact across individual, unit organizational and system levels, as captured in the developed multi‐level framework (see Figure 5). This explains why one single uncoordinated approach will have limited impact. For example, holding a research workshop without protected time, leadership support, research mentorship and management approval will have a limited impact on fostering an organizational culture of nursing excellence. Facilitators at all four system levels must be activated for success.

Within nursing research, a paradox of recognition versus action exists among healthcare organizations. Nurses are eager and willing to engage in clinical research and organizations recognise its value; however, many barriers remain, with limited opportunities for nurses. Nurses from an academic hospital in the United States (*N* = 99) reported that they had a high willingness to engage in research‐related tasks (Nowlin et al. 2021). However, they also reported that they had low to moderate knowledge and ability to perform research (Nowlin et al. 2021). In a study in Norway of clinical nurses from a university hospital (*N* = 364), 40% had positive attitudes towards research; however, few were engaged in research‐based activities (Akerjordet et al. 2012a). In yet another cross‐sectional study in the United States, oncology nurses (*N* = 623) reported positive attitudes towards nursing research and believed research should guide their practice. However, they reported significant barriers to engaging in research, including time, knowledge and lack of resources (Arthur et al. 2019). While nurses have a clear appreciation and eagerness towards nursing research, it remains a reality that most are not given the opportunity to conduct research. This is detrimental to patient care because nurses' ability to engage in research is directly related to the safety and quality of care they provide to patients and families (Harding et al. 2016; Ives Erickson and Pappas 2020).

In this scoping review, time was a reported barrier to nurses engaging in research in 51 of the 79 included articles. Given that nurses are eager and willing to conduct research, it is most likely a lack of time that prevents them from conducting clinical research. For many nurses, it is expected that they conduct research in their own time and unpaid, which results in fewer nurses conducting research. The second most frequently reported barrier to nurses' engagement in research, reported in 30 of the included articles, was a widespread lack of research knowledge and skills. One reason for this was insufficient mentorship. Most undergraduate nursing programmes offer nurses one course on EBP, where they are taught to be consumers of research (Coyne et al. 2018). However, they are rarely taught how to conduct sound clinical research. Then, after completion of their bachelor's degree in nursing, they enter clinical roles where they do not engage in research or generate evidence; therefore, any research skills they learned during their education wane. As demonstrated in the findings of this review, visionary leadership and the development and implementation of innovative nursing programmes and interventions are significant ways to increase the generation of nursing evidence within a healthcare organization. This is because these solutions directly combat the two main barriers: they afford nurses paid clinical release time and education and training on research.

Globally, nurses are experiencing moderate to high levels of burnout syndrome (Ge et al. 2023), healthcare organizations are faced with nursing shortages (Muir et al. 2024) and high levels of nurses are exiting the profession (Mulisa et al. 2022). However, there is a solution that leaders of healthcare organizations can use to combat burnout and nursing shortages: promoting a culture of nursing research and innovation by increasing the generation of nursing evidence. This is because when healthcare organizations promote and prioritise nursing research, nurses have significantly increased levels of work satisfaction, are more loyal to the organization and are retained (Ives Erickson and Pappas 2020). One hospital implemented a research‐focused academic‐service partnership to increase research capacity and research productivity (Duffy et al. 2015). After 1 year, research productivity within the hospital increased 33%. Therefore, there is a practical solution to improve the quality and safety of nursing care and to address nursing burnout and shortages: increasing nurses' ability to engage in research and generation new updated evidence. Leaders are in a position to shift the organizational culture to normalise and value nursing research. This scoping review highlights facilitators that leaders of healthcare organizations can utilise to do just that.

## 9 Strengths and Limitations

Several limitations should be acknowledged in this scoping review. First, articles were included if they were published in English from 2010 onwards; therefore, relevant articles published in other languages before 2010 would have been excluded. Grey literature was excluded, which could have resulted in the omission of relevant information. The geographic context of included articles was limited to developed countries; therefore, the findings cannot be generalized to low‐ and middle‐income countries.

There are strengths worth noting in this scoping review. First, it followed the established JBI methodology. The search strategy was conducted using a comprehensive process, titles and abstracts were screened by two independent reviewers and all data was extracted by one reviewer and checked by another. A wide range of article types was included, which resulted in a robust number of included articles, and a comprehensive overview of the multiple barriers and facilitators to clinical nurses engaging in research.

## 10 Conclusion

Findings from this scoping review provided an overview of the factors that influence clinical nurses' engagement in research. Multiple barriers were identified that prevented nurses from engaging in research, including a lack of paid release time, training and mentorship. However, multiple facilitators were identified that enabled nurses to engage in research, including visionary leadership and the development of programmes and centres that foster a culture of research excellence. A multi‐level fromaework that depicts how barriers and facilitators at four levels (i.e., individual, unit, organizational, and system) influence nurses engagement in research. Leaders and decision‐makers within healthcare organizations can use this framework to understand the barriers that prevent nurses from engaging in research and utilise the facilitators to advance nursing research in clinical settings. Developing a culture of nursing research excellence within healthcare organizations that empowers nurses to generate research evidence will promote nurses' professional development, ensure safe, high‐quality nursing care and help sustain the nursing workforce.

## Author Contributions

Lyndsay Jerusha MacKay: conceptualization, data curation, formal analysis, funding acquisition, investigation, methodology, project administration, supervision, visualization, writing original draft, writing review and editing. Laurie Lee: conceptualization, formal analysis, funding acquisition, methodology, writing review and editing. Suzanna Crawford: conceptualization, funding acquisition, writing review and editing. K. Alix Hayden: conceptualization, investigation, methodology, writing review and editing. Gabrielle Harrison: data curation, formal analysis, writing review and editing. Jessica Son: data curation, writing review and editing. Josuha Yudkin: methodology, formal analysis, writing review and editing. Sara Moore: formal analysis, writing review and editing.

## Funding

This work was supported by Trinity Western University's Undergraduate Student Research Award (USRA) and University of Calgary's Faculty of Nursing sponsored this project.

## Conflicts of Interest

The authors declare no conflicts of interest.

## Supporting information


**File S1:** Preferred reporting items for systematic reviews and meta‐analyses extension for scoping reviews (PRISMA‐ScR) Checklist.

**File S2:** Final searches conducted in: ovid MEDLINE(R), Embase, APA Psychinfo, CINAHL plus with full text (EBSCO), Scopus.

**File S3:** 37 developed nations as per the United Nations Department of Economic and Social Affairs.

## Data Availability

The data that support the findings of this study are available from the corresponding author upon reasonable request.
