# Supplementary material for: Strengthening Clinical Nurses' Research Capacity: A Scoping Review of Factors That Influence Nurses' Engagement in Clinical Research
Source: J Clin Nurs. 2026 Jun 19;35(11):4582–628. doi: 10.1111/jocn.70401 (PMC13629278; doi:10.1111/jocn.70401)
Supplement: Supplementary file 1 [file JOCN-35-4582-s001.docx]

**Supplementary File 2**

**FINAL Searches**

**Review:**

# **Exploration of clinical nurses’ engagement in research and evidence-based practice: A scoping review**

**Date: April 28, 2025**

Database(s): **Ovid MEDLINE(R) ALL** 1946 to April 25, 2025
Search Strategy:

| **#** | **Searches** | **Results** |
| --- | --- | --- |
| 1 | (nurse* adj4 (engag* or participat* or utiliz* or utilis* or capacity or commitment or aware* or literacy or interact* or access* or reflect* or generat* or involv* or attitude* or led or lead or leading or "use" or "using") adj5 ("evidence based" or "evidence informed" or EBP or research)).tw,kf. | 2444 |
| 2 | ((engag* or participat* or utiliz* or utilis* or capacity or commitment or aware* or literacy or interact* or access* or reflect* or generat* or involv* or attitude* or led or lead or leading or "use" or "using") adj5 ("evidence based" or "evidence informed" or EBP or research) adj4 nurse*).tw,kf. | 2384 |
| 3 | 1 or 2 | 2729 |

Database(s): **Embase** 1974 to 2025 April 25
Search Strategy:

| **#** | **Searches** | **Results** |
| --- | --- | --- |
| 1 | (nurse* adj4 (engag* or participat* or utiliz* or utilis* or capacity or commitment or aware* or literacy or interact* or access* or reflect* or generat* or involv* or attitude* or led or lead or leading or "use" or "using") adj5 ("evidence based" or "evidence informed" or EBP or research)).tw,kf. | 2717 |
| 2 | ((engag* or participat* or utiliz* or utilis* or capacity or commitment or aware* or literacy or interact* or access* or reflect* or generat* or involv* or attitude* or led or lead or leading or "use" or "using") adj5 ("evidence based" or "evidence informed" or EBP or research) adj4 nurse*).tw,kf. | 2684 |
| 3 | 1 or 2 | 3049 |
| 4 | limit 3 to conference abstract | 402 |
| 5 | 3 not 4 | 2647 |

Database(s): **APA PsycInfo** 1806 to April 2025 Week 3
Search Strategy:

| **#** | **Searches** | **Results** |
| --- | --- | --- |
| 1 | (nurse* adj4 (engag* or participat* or utiliz* or utilis* or capacity or commitment or aware* or literacy or interact* or access* or reflect* or generat* or involv* or attitude* or led or lead or leading or "use" or "using") adj5 ("evidence based" or "evidence informed" or EBP or research)).ti,ab,id. | 864 |
| 2 | ((engag* or participat* or utiliz* or utilis* or capacity or commitment or aware* or literacy or interact* or access* or reflect* or generat* or involv* or attitude* or led or lead or leading or "use" or "using") adj5 ("evidence based" or "evidence informed" or EBP or research) adj4 nurse*).ti,ab,id. | 843 |
| 3 | 1 or 2 | 980 |

**CINAHL Plus with Full Text (Ebsco)**

Search modes - Find all my search terms

| **#** | **Query** | **Results** |
| --- | --- | --- |
| S1 | TI ( (nurse* N3 (engag* or participat* or utiliz* or utilis* or capacity or commitment or aware* or literacy or interact* or access* or reflect* or generat* or involv* or attitude* or led or lead or leading or "use" or "using") N4 ("evidence based" or "evidence informed" or EBP or research)) ) OR AB ( (nurse* N3 (engag* or participat* or utiliz* or utilis* or capacity or commitment or aware* or literacy or interact* or access* or reflect* or generat* or involv* or attitude* or led or lead or leading or "use" or "using") N4 ("evidence based" or "evidence informed" or EBP or research)) ) | 2,900 |
| S2 | TI ( ((engag* or participat* or utiliz* or utilis* or capacity or commitment or aware* or literacy or interact* or access* or reflect* or generat* or involv* or attitude* or led or lead or leading or "use" or "using") N4 ("evidence based" or "evidence informed" or EBP or research) N3 nurse*) ) OR AB ( ((engag* or participat* or utiliz* or utilis* or capacity or commitment or aware* or literacy or interact* or access* or reflect* or generat* or involv* or attitude* or led or lead or leading or "use" or "using") N4 ("evidence based" or "evidence informed" or EBP or research) N3 nurse*) ) | 2,845 |
| S3 | S1 OR S2 | 3,258 |

**Scopus**

N=1437

( TITLE ( ( nurse* W/3 ( engag* OR participat* OR utiliz* OR utilis* OR capacity OR commitment OR aware* OR literacy OR interact* OR access* OR reflect* OR generat* OR involv* OR attitude* OR led OR lead OR leading OR "use" OR "using" ) W/4 ( "evidence based" OR "evidence informed" OR ebp OR research ) ) ) OR ABS ( ( nurse* W/3 ( engag* OR participat* OR utiliz* OR utilis* OR capacity OR commitment OR aware* OR literacy OR interact* OR access* OR reflect* OR generat* OR involv* OR attitude* OR led OR lead OR leading OR "use" OR "using" ) W/4 ( "evidence based" OR "evidence informed" OR ebp OR research ) ) ) OR TITLE ( ( ( engag* OR participat* OR utiliz* OR utilis* OR capacity OR commitment OR aware* OR literacy OR interact* OR access* OR reflect* OR generat* OR involv* OR attitude* OR led OR lead OR leading OR "use" OR "using" ) W/4 ( "evidence based" OR "evidence informed" OR ebp OR research ) W/3 nurse* ) ) OR ABS ( ( ( engag* OR participat* OR utiliz* OR utilis* OR capacity OR commitment OR aware* OR literacy OR interact* OR access* OR reflect* OR generat* OR involv* OR attitude* OR led OR lead OR leading OR "use" OR "using" ) W/4 ( "evidence based" OR "evidence informed"OR ebp OR research ) W/3 nurse* ) ) ) AND NOT INDEX ( medline ) AND NOT ( PMID ( 0* OR 1* OR 2* OR 3* OR 4* OR 5* OR 6* OR 7* OR 8* OR 9* ) ) AND NOT INDEX ( embase )

NOTE: Excluded Medline, Embase and PubMed records

<https://searchfilters.cda-amc.ca/link/97>

Citation: Scopus NOT Medline/PubMed NOT Embase - Scopus. In: Canada's Drug Agency Search Filters Database. Ottawa: Canada's Drug Agency; 2025: <https://searchfilters.cda-amc.ca/link/97>. Accessed 2025-04-28.
